# Exploring lightweight knowledge distillation with adversarial attack for efficient coronary artery vessel segmentation

**DOI:** 10.3389/frai.2026.1891993

**Published:** 2026-10-30

**Authors:** Hao Dang, Xuan Nie, Shipei Jing, Peng Li

**Affiliations:** 1 School of Information and Technology, Henan University of Chinese Medicine, Zhengzhou, China; 2 Zhengzhou Key Laboratory for Intelligence Analysis and Utilization of Chinese Medicine Information, Henan University of Chinese Medicine, Zhengzhou, China; 3 School of Software, Northwestern Polytechnical University, Xi'an, China; 4 School of Information and Engineering, Henan University of Animal Husbandry and Economy, Zhengzhou, China; 5 Institute for Complexity Science, Henan University of Technology, Zhengzhou, China

**Keywords:** adversarial attack, attention mechanism, coronary artery segmentation, deep learning, knowledge distillation

## Abstract

**Introduction:**

Vessel segmentation is significant for diagnosing coronary atherosclerotic heart disease. Although deep learning has achieved remarkable progression coronary vessel segmentation, existing deep learning-based methods still suffer from huge computational costs, overfitting in small sample scenarios, and traditional loss functions that fail to balance class imbalance and vascular topological continuity.

**Method:**

To address these limitations, we propose KDLight, a novel and lightweight knowledge distillation framework with adversarial attack designed for coronary artery vessel segmentation. The novel framework include the three components of adversarial attacker module, generator and knowledge distillation module. Specifically, we first embed an adversarial attack module with black-box attacker before the generator. It is capable of producing meaningful perturbations and adversarial examples throughout the training process, which is beneficial for enhancing the generalization ability and decreasing over-fitting of model. The additional knowledge distillation module consists of the teacher and student refinement sub- network. The feature-level similarity distillation module and pixel-level similarity distillation module are respectively implemented to feature extraction and vessel segmentation. Different from existing knowledge distillation paradigms, our teacher module is a segmentation network that incorporates more inputs to effectively capture noise distributions and further refine segmentation predictions during training. The acquired knowledge is subsequently transferred to the student module via feature- level similarity distillation module, empowering the student to produce improved segmentation outputs.

**Results:**

Extensive experiments conducted on Dataset 1 and Dataset 2 indicate that LightKD achieves superior generalization capabilities and outperforms state-of-the-art approaches in various coronary vessel segmentation tasks. Critically, these performance improvements are statistically confirmed by consistent *p*-values less than 0.05.

**Discussion:**

Comparative experiments with recent state-of-the-art knowledge distillation segmentation methods show that KDLight attains competitive accuracy at markedly lower FLOPs and parameter counts. Moreover, the pipeline generalizes well across multiple student backbones, confirming that it can be migrated to different lightweight encoder architectures. In future work, we will collect additional multi-center angiography data to mitigate the domain gap and further improve cross-dataset generalization.

## 1 Introduction

Coronary artery disease (CAD), manifested as stenosis or occlusion of coronary arteries, represents one of the most prevalent cardiovascular disorders and has remained the primary global cause of mortality over the last two decades (Yang et al., 2026). Currently, X-ray coronary angiography (XCA) serves as the clinical gold standard for evaluating the anatomical degree of stenosis, owing to its capability to clearly visualize coronary arteries and their branching structures (Collet et al., 2017; Tavakol et al., 2012). Physicians can quantitatively and qualitatively assess the location, scope, and severity of vascular lesions, as well as vessel morphological features, based on angiographic findings (Chen et al., 2026). Consequently, accurate segmentation of vascular structures from coronary angiograms is crucial for disease screening and clinical diagnosis.

Segmentation of coronary arteries from continuous XCA sequences demands temporal consistency across consecutive frames. However, the segmentation of coronary artery vessel still embraces challenges as illustrated in Figure 1. Firstly, cross-frame localization of the vessel suffers from inherent ambiguity. Cardiac vessel motion and similar morphological features cause misidentification between main trunks and branch vessels (Tang et al., 2025; Zhang et al., 2022). Secondly, the XCA images are seriously disturbed by high-frequency noise and dynamic interference (Fu et al., 2024). For vessel segmentation, it leads to inaccurate segmentation results.

**Figure 1:**
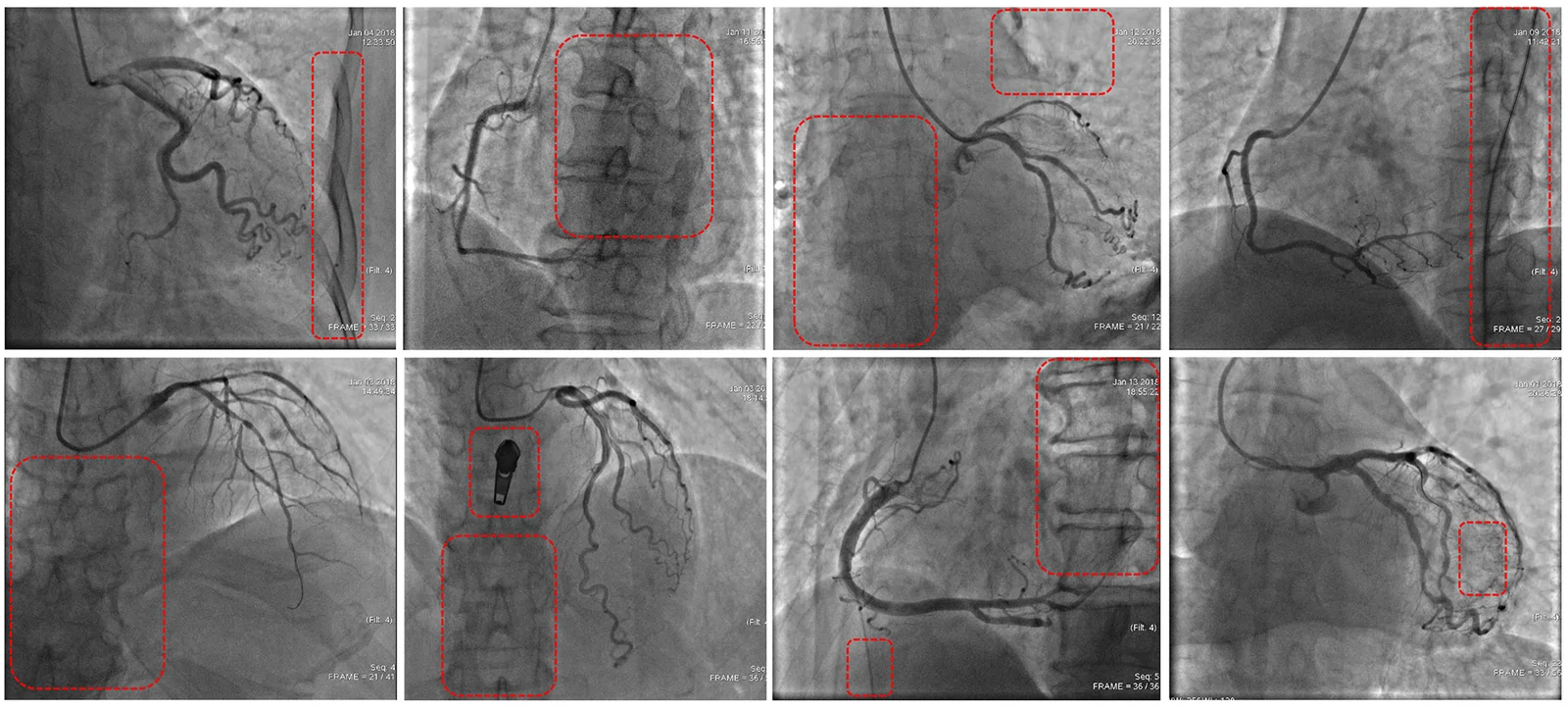
The coronary angiographic image specimens. Red bounding boxes denote the complex background and noise of angiography images. Zoom in for better details.

Traditional image processing methods has required manual image segmentation by experienced professionals. It is a process that is both time-consuming and subject to human error, which suffers from large computational costs, coarse segmentation results, and poor adaptability to variations in image quality. Currently, all proposed XCA vessel segmentation methods based on single-frame vessel segmentation have been proposed for evaluating the severity of stenosis (Avram et al., 2023; Pang et al., 2021; Zhang D. et al., 2024; Zhang X. et al., 2024), the assessment of coronary blood flow still relies on semi-manual interaction, which are time-consuming and observer-dependent (Zhang et al., 2019). To address these challenges, automated coronary segmentation technologies have emerged as a major focus within the field of computer-aided medical diagnosis.

In recent years, deep learning technology has achieved outstanding results in coronary vessel segmentation. Particularly, the convolutional neural network (CNN) architecture exemplified by UNet (Ronneberger et al., 2015) have breathed new life into the field of automatic vascular segmentation. Liu et al. (2022) proposed FR-UNet, which integrates fullresolution network and dual-threshold iteration for retinal vessel and coronary angiograph segmentation. Priyadarsini et al. (2023) further explored UNet++ (Zhou et al., 2018) for retinal vessel segmentation. Currently, the attention mechanism has been widely applied to vessel segmentation tasks. For example, DenseNet-Attention-U-Net (Liang et al., 2020) combined DenseNet, a convolutional block attention module, and U-Net for retinal vessel segmentation. Guo et al. (2021) introduced SA-UNet, which incorporates a spatial attention module and replaces traditional convolutional blocks with structured dropout convolutional blocks to mitigate network overfitting. Despite the progress achieved in XCA semantic segmentation, existing research fails to realize the discrimination and identification of independent coronary vessels. The coronary arterial tree constitutes an intricate anatomical architecture formed by the left coronary artery (LCA) and right coronary artery (RCA). Both arteries branch into a large number of subsegments with unique morphological traits. Otherwise, these methods suffer from excessive computational overhead and overfitting.

To overcome these limitations, this paper presents a lightweight knowledge distillation framework with adversarial attack, namely KDLight, for coronary vessel segmentation task. The framework includes the generator, a knowledge distillation network attached to it and an adversarial attacker ahead of it, as shown in Figure 2. We leverage the adversarial attacker to generate subtle perturbations for input data. Both the perturbed and original samples are jointly utilized for model training. Meanwhile, the real-time performance feedback derived from the knowledge distillation network further enhances the robustness of model during optimization. The knowledge distillation network leverages semantic feature-level similarity and pixel-level similarity to segment coronary arteries from XCA images.

**Figure 2:**
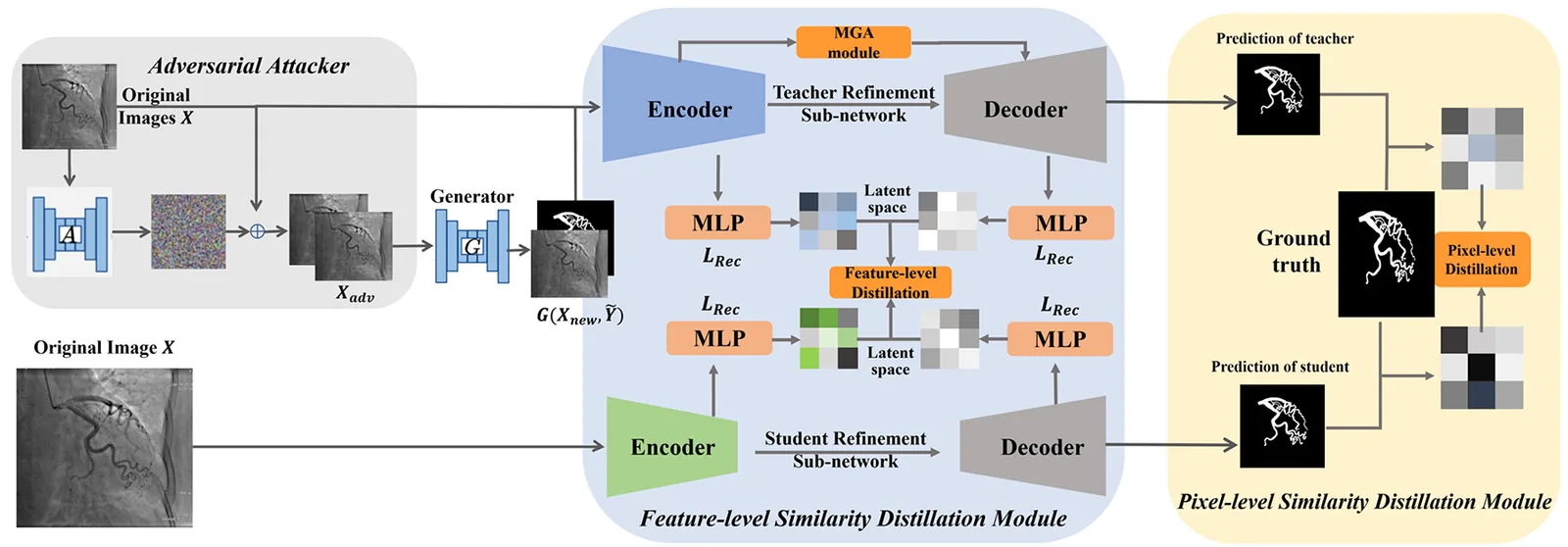
The framework of our proposed SKD-Net. The MGA module generates more refined pixel-level attention maps from high-level features, thus enhancing the modeling of long-range dependencies. The FSD module performs feature distillation, which focuses on transferring structural knowledge from a complex teacher network to a lightweight student network. Meanwhile, the ASD module implements adversarial distillation to measure the similarity between model predictions and labels.

The main contributions of this work are summarized as follows:

- We propose KDLight Vessel, a novel framework capable of achieving temporally consistent segmentation of the coronary artery vessels from XCA images, offering a valuable tool for coronary artery disease diagnosis. It include the three components of adversarial attacker, generator and knowledge distillation network.
- We introduce a black-box adversarial attacker that augments training data with adversarial perturbations guided by feedback from the generator, thereby effectively improving the overall robustness of the segmentation model.
- The knowledge distillation network integrates semantic feature-level similarity and pixel-level similarity to transmit systematic knowledge from a complex teacher to the lightweight student. In teacher refinement sub-network, the Multi-Scale Group Attention Module (MGA) is utilized to combine multi-scale cross-channel and cross-spatial contextual semantic information. This module focuses on retaining per-channel information while reducing computational overhead.
- The feature-level similarity distillation module (FSD) is implemented to models semantic shifts by calculating feature similarity across symmetric encoder-decoder layers. It obtain the semantic similarity of feature-level. The pixel-level similarity distillation (PSD) module guides the student network to explore fine-grained semantic knowledge by measuring pixel-level correlations between the teacher–student predictions and ground-truth annotations.
- Extensive experiments are conducted on the PCAD and the DCA1 datesets. Comparing KDLight Vessel with advanced approaches, our presented new method has achieved good performance in the task of coronary vessel segmentation.

## 2 Related work

### 2.1 Knowledge distillation in medicine image segmentation

Deep learning-based have achieved remarkable achievements in heart disease prediction (Anitha et al., 2025; Duraisamy et al., 2025; Madhusundar et al., 2025a,b). Knowledge distillation (Hinton et al., 2015) is an effective technique that transfers latent knowledge from large-scale, sophisticated teacher network to compact student network. This strategy substantially boosts the performance of student while incurring negligible extra memory and computational overhead (Habib, 2024; Gou et al., 2023). In medical imaging, although knowledge distillation exhibits promising prospects this technique still remains insufficiently investigated (Zhang et al., 2025). Javed (2023) proposed a knowledge distillation algorithm to improve the performance of shallow networks for tissue phenotyping in histology images. Xie et al. (2023) proposed deep mutual distillation (DMD), a high-entropy online mutual distillation process that enhances semi-supervised medical image segmentation by providing more informative training than low-entropy methods, particularly in ambiguous regions. To tackle the multi-class label imbalance challenge, You et al. (2023) proposed an anatomical-aware contrastive distillation framework for semi-supervised medical image segmentation, which leverages iterative contrastive distillation, soft negative labeling, and anatomical contrast sampling to boost segmentation accuracy.

### 2.2 Attention mechanisms in medicine image segmentation

Research on attention mechanisms for coronary artery vessel segmentation can be categorized into three categories: channel attention, spatial attention, and combined channel and spatial attention. Channel attention allows the model to focus attention on different channels of the input data, enabling it to better capture key information from each channel (Yang and Qi, 2021). Xiao et al. (2025) proposed RDTransUNet, a hybrid U-Net architecture that integrates residual connections with dual channel–spatial attention mechanisms, aiming to jointly capture local details and global contextual information for medical image segmentation. Quan et al. (Quan and Liatsis, 2026) proposed a lightweight U-Net backbone for 3D coronary vessel segmentation, explicitly designed to achieve a favorable trade off between segmentation accuracy and GPU memory consumption. (Zhang M. et al. 2024) designed a context interactive deep network specifically for coronary vessel segmentation, integrating a bioinspired attention block and a multiscale interactive block to optimize the capture of spatial and vessel edge information. Wu et al. (2025) proposed a multi-attention dynamic sampling network Multi-ADS-Net and built a crossdataset pre-trained Multi-ADS-Net model for generalizable vessel segmentation in CAG images.

### 2.3 Adversarial attacker in medicine image segmentation

There are usually two types of adversarial attacks, white-box attack and black-box attack. The former attacker can fully access the target model including model structure and parameters, while the latter attacker has little knowledge of the target model (Zhao et al., 2025). Narodytska and Kasiviswanathan (2017) first proposed black-box attack method. The target network was approximated by querying output labels to train a surrogate model that generates adversarial examples for the original network. Xia et al. (2024) proposed a method named DiffDistill for multi-contrast medical image translation on MRI datasets, which combines Diffusion models with knowledge distillation. Most existing attack strategies are based on white-box training by specifying attack parameters and pre-trained models to generate gradient-based adversarial examples, which lack flexibility and may limit generalization capability. On the other hand, black-box training only focuses on training models using adversarial examples, which may be corrupted and thus well explores the model performance on perturbed images. In this paper, a black-box attacker is designed to generate adversarial examples through real-time performance feedback of the target model. It produces more meaningful disturbances through the feedback from the integrated generator, and the produced perturbation data further help the learn of image segmentation.

## 3 Methods

### 3.1 Overview

The full overview of our workflow is illustrated in Figure 2, which includes four parts: adversarial attacker module, generator, feature-level similarity distillation module (FSD), and pixel-level similarity distillation module (PSD). First, we use the adversarial attacker to introduce certain perturbations θ to the original input images *X*∈ℝ^*W* × *H*^ and use them together with the original data as the training set, while its robustness can be improved by real-time performance feedback of the knowledge distillation network. The adversarial attacker generates adversarial samples $X_{adv}\in {\mathbb{R}}_{adv}^{W\;\times \;H}$. The middle part is the embedded *generator G*, which can be any kind of generators. The *generator G* generates the new samples and their labels *G*(*X*_*new*_, Ỹ) based on *X*_*adv*_. Then the new samples *G*(*X*_*new*_, Ỹ) and augmented original images *I*(*X, Y*) are fed into the distillation network. The knowledge distillation network includes the feature-level similarity distillation module and pixel-level similarity distillation module, which can obtain improved fine-grained semantic information from feature-level and pixel-level.

### 3.2 Adversarial attacker

We utilize a black-box attacker F to generate adversarial examples, which is intended to reflect the vulnerability of the segmentation network, and the network continuously improves its robustness through current feedback to adapt to this attack. We generate *X*_*adv*_ by adding visible adversarial perturbations to each original image *X*. Specifically, the adversarial perturbations include four types: Gaussian, uniform, impulse and misalignment as depicted in Figure 3. For this purpose, a *generator* *G* with condition θ is designed as the attacker to generate perturbations, It is defined as Equation 1.


$$\begin{array}{l} X_{adv}=X+F\left(X,\theta \right) \end{array}$$ (1)

**Figure 3:**
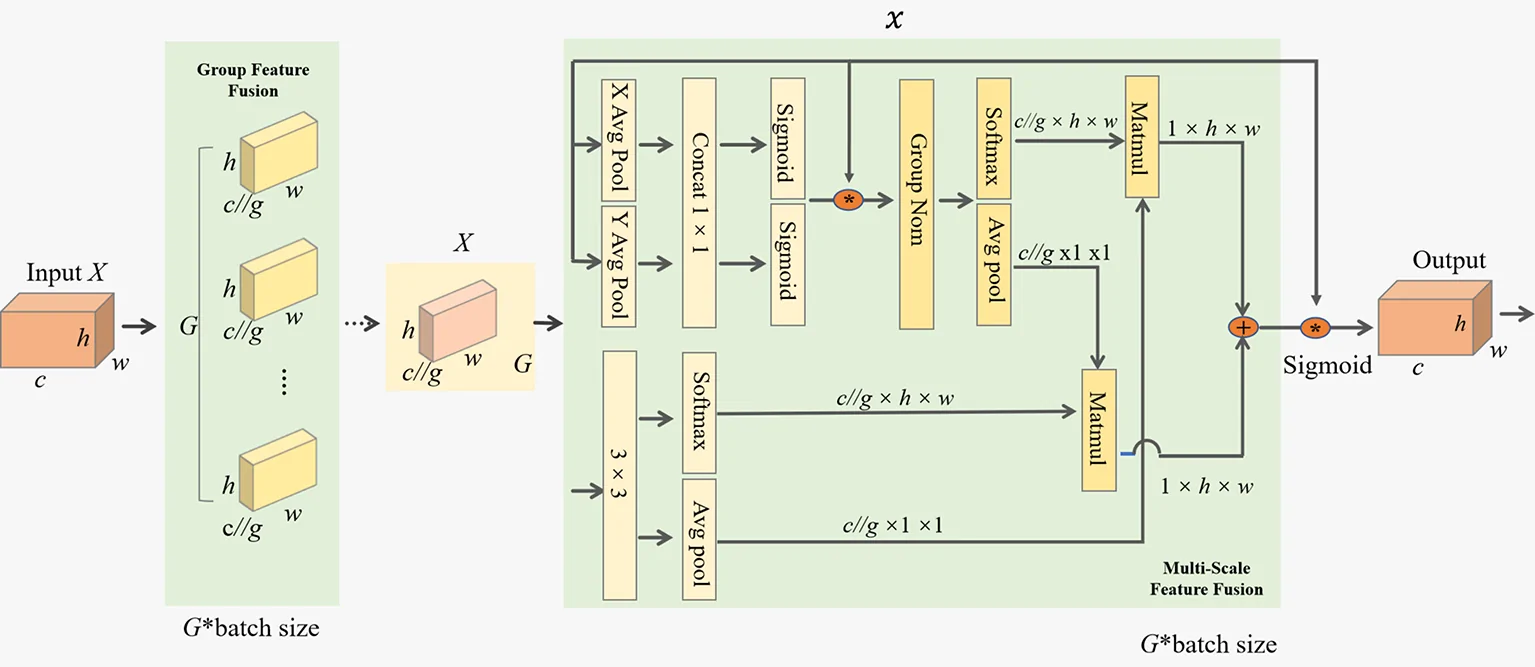
Illustration of the multi-scale group attention module. Here, *G* denotes the number of divided groups, *X Avg Pool* refers to the one-dimensional horizontal global average pooling, and *Y Avg Pool* represents the one-dimensional vertical global average pooling, respectively. *Matmul* means Matrix Multiplication.

where θ represents the noise such as the Gaussian, uniform, impulse and misalignment perturbations. Adding these types of noise to input data helps simulate real-world effects from factors such as sensor imperfections, environmental conditions, transmission errors, and patient movement, which can all impact the quality and accuracy of medical imaging. F(X,θ) is a U-net with input *X* and noise θ, and its output is an image of noise with the same size of *X*.

The purpose of generated adversarial examples *X*_*adv*_ is to interfere with the *generator* *G*. The feedback from the *generator* during the training process allows the attacker F to independently learn and refine its performance, resulting the high robustness of the segmentation network. This is an iterative process of continually optimizing the attacker, controlled by minimizing the loss functions $L_{atk}\left(2\right)$.

### 3.3 Generator based on adversarial attacker

The black-box attacker generates adversarial examples $X_{adv}\in {\mathbb{R}}_{adv}^{W\;\times \;H}$. Then the *generator* *G*(*X*_*new*_, Ỹ) generates new examples *X*_*new*_ and their labels Ỹ according to adversarial examples *X*_*adv*_, *G*(*X*_*new*_, Ỹ) = *G*_*new*_(*G*(*X*_*adv*_), Ỹ). To ensure the robustness of *G*(*X*_*new*_, Ỹ), we introduce the attack loss $L_{atk}$ to encourage the attacker to generate perturbations that are appropriate for the input image based on the current feedback. It is defined as Equation 2:


$$\begin{array}{l} L_{atk}=\frac{1}{{𝔼}_{X_{new}}\left[{||Ỹ-G\left(X_{adv}\right)⚬G_{new}\left(G\left(X_{adv}\right),Ỹ\right)||}_{1}\right]+\varepsilon } \end{array}$$ (2)

When *generator* *G* is able to learn the characteristics of the adversarial examples and process them correctly, the attacker A generates more difficult examples. This adversarial process not only prevents *G* from overfitting but also enhances the robustness of the model for image segmentation. The relaxation factor ε = 0.1 is introduced to constrain the change in the loss value within a reasonable interval, avoiding over-attack.

The adversarial loss $L_{adv}$ in traditional adversarial process is applied between a *generator* *G* and a *discriminator D*, and It is defined as Equation 3:


$$\begin{array}{l} L_{adv}\left(G,D\right)={𝔼}_{Ỹ}\left[log\left(D\left(Ỹ\right)\right)\right]+{𝔼}_{X}\left[log\left(1-D\left(G\left(X\right)\right)\right)\right] \end{array}$$ (3)

Based on the loss of $L_{atk}$ and $L_{adv}$, the objective of our pre-training phase of adversarial attacker is shown as follows considering other related loss functions and their corresponding weights:


$$\begin{array}{l} \underset{G,G_{new}}{\text{min}}\underset{D}{\text{max}}L_{o}(G,G_{new},D)=\lambda L_{atk}+\mu L_{adv} \end{array}$$ (4)

where λ and μ are two hyper-parameters, which balance the loss of $L_{atk}$ and $L_{adv}$.

### 3.4 Knowledge distillation base on refinement

The added refinement sub-networks is constructed using knowledge distillation, which includes the teacher and student refinement sub-networks with different structures and inputs to refine the performance of segmentation. As shown in Figure 2, the teacher refinement sub-network learns more knowledge in pre-training stage and then transfers the knowledge to the student refinement sub-network.

#### 3.4.1 Teacher refinement sub-network

The more inputs of combining original and generated images (*X and X*_*new*_) are fed into our teacher refinement sub-network with multi-Scale group attention module (MGA), which is based on a network and composed of U-net network and Selective Kernel block (SKblock). Concretely, we constructs a parameter-intensive teacher refinement sub-network based on the U-Net (Ronneberger et al., 2015) backbone, in which SKblock based on multi-Scale group attention module (MGA) are hierarchically stacked within the encoder. In SKBlock, the network simultaneously processes the input using multiple convolutional kernels of different sizes (3 × 1, 5 × 1, 7 × 1, 9 × 1) to generate feature representations at various scales. The knowledge from parameter-intensive teacher refinement sub-network is transferred by feature-level similarity distillation module (FSD).

##### 3.4.1.1 Multi-scale group attention module

To model the cross-channel relationships without performing channel dimensionality reduction and while reducing the computational overhead, we embed an MGA module into the feature mapping process from encoder to decoder. Specifically, by reshaping partial channels into the batch dimension and dividing the channel dimension into several sub-features, we facilitate better allocation of spatial semantic representations for intricate coronary artery vessels across different feature groups. This grouping strategy effectively preserves the integrity of spatial semantics while facilitating the capture of fine-grained inter-channel dependencies. It achieves a cross-channel and cross-spatial features interaction by two parallel group networks. As illustrated in Figure 4, MGA includes multi-scale feature integration and group feature fusion. Considering the multi-scale and feature group architecture, this is advantageous for efficiently capturing short-term and long-range dependencies, thereby enhancing overall performance.

**Figure 4:**
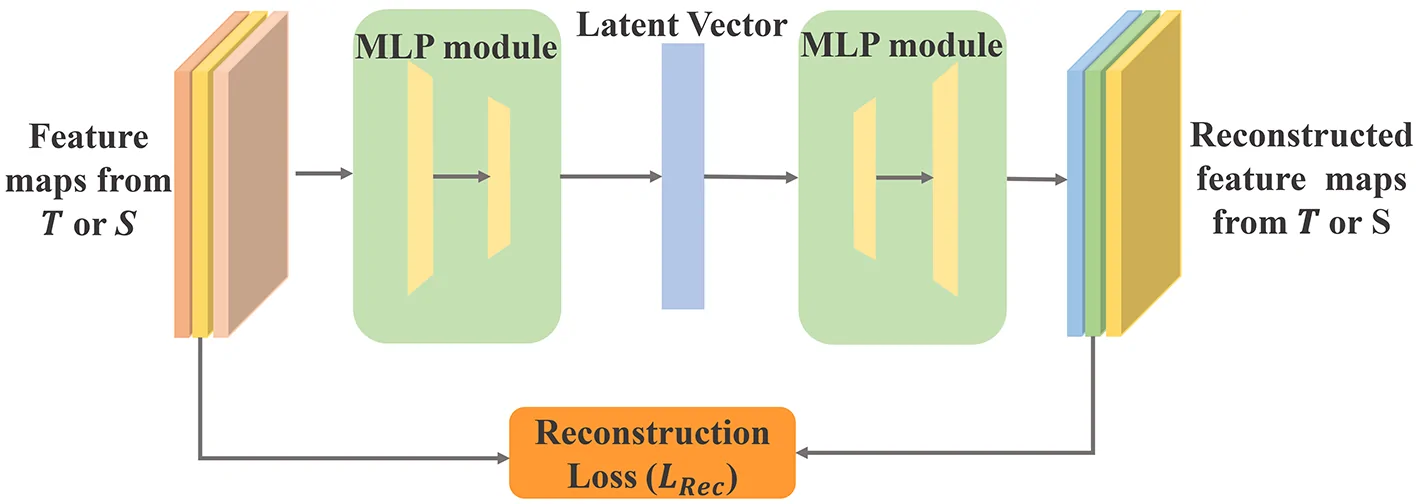
The structure of the MLP module. The contextual feature maps are mapped into a latent feature space via two multi-layer perceptron (MLP) with GELU activation, which performs linear projection, non-linear transformation, and a second linear projection. The MLP module is constructed to generate latent vectors. Finally, the semantic feature information before and after the hidden vector is reconstructed via the reconstruction loss $L_{Rce}$.

##### 3.4.1.1.1 Group feature fusion

As illustrated in Figure 4, for given original input feature maps *X*∈ℝ^*C* × *W* × *H*^ and generated new samples $X_{new}\in {\mathbb{R}}_{new}^{C\;\times \;W\;\times \;H}$, the proposed MGA module first partitions *X* and *X*_*new*_ into *K* = [*k*_0_, *k*_1_, ⋯ , *k*_*G*−1_] (*K*<*C*) groups across the channel dimensions direction, where each group is dedicated to learning distinct semantic information. The grouping strategy is formally defined by *X* = [*x*_0_, *x*_1_, ⋯ , *x*_*K*−1_] and $X_{new}=\left[x_{new}^{1},x_{new}^{2},\cdots \;,x_{new}^{K-1}\right]$, which determines the partition of channel dimensions to ensure that semantically relevant channels are grouped. After grouping, attention weights are adaptively computed and assigned to each group, which serves to strengthen the feature representations of region-of-interest (ROI) within each group while suppressing irrelevant background features. This group-wise attention mechanism enables the MGA module to focus on critical semantic information without introducing redundant computations, thereby enhancing the discriminability of feature representations for coronary artery vessel segmentation.

##### 3.4.1.1.2 Multi-scale feature fusion

Notably, the large receptive fields allow for efficient aggregation of multi-scale spatial contextual features. Inspired by the inherent advantage, we exploit three parallel pipelines to derive the descriptors of attention weights corresponding to multi-scale features, thereby facilitating the capture of hierarchical spatial semantics across different scales. The architecture consists of three parallel pipes: two branches adopt 1 × 1 convolutions, while the remaining one uses 3 × 3 convolutions. To efficiently model cross-channel dependencies with low computational overhead, we introduce a channel-wise information interaction mechanism. Specifically, in two 1 × 1 branches, we conduct two independent one-dimensional global average pooling along the respective spatial dimensions (*X* and *X*_*new*_). Concurrently, the 3 × 3 branch employs a one-fold 3 × 3 kernel to extract multi-scale information, which enables the module to aggregate both local and contextual information across different spatial scales efficiently. The Multi-scale feature fusion integrates the cross-channel and cross-spatial attentions.

##### 3.4.1.2 Feature-level similarity distillation module

The FSD module is proposed as a principled feature distillation paradigm, dedicated to transfer structured contextual knowledge and alignment feature distribution from a high-capacity and parameter-intensive teacher refinement sub-network *T* to a compact and parameter-efficient student refinement sub-network *S*. The essential optimization goal is to compress the multi-scale hierarchical contextual priors, discriminative semantic patterns, and implicit feature knowledge embedded in the well-trained teacher network into the lightweight student network, which realizes efficient model parameter compression and computational acceleration while mitigating feature representation degradation. Concretely, *T* and *S* hold heterogeneous architectural configurations, leading to inherent mismatches in feature dimension. Such structural and statistical discrepancies prohibit direct feature-level distillation and cross-network knowledge matching. To resolve this dimensional and distribution inconsistency, we first deploy a lightweight multi-layer perceptron (MLP) as a shared projection mapping function. As visualized in Figure 5, this MLP conducts a bi-directional feature projection from encoder and decoder, transforming the heterogeneous high-dimensional feature tensors of both teacher and student networks into a unified low-dimensional latent embedding space. This canonical feature calibration mechanism enables rigorous and stable homogeneous knowledge distillation across heterogeneous architectures, compelling the student refinement sub-network to implicitly approximate the feature mapping function and hierarchical representation learning paradigm of the teacher refinement sub-network.

**Figure 5:**
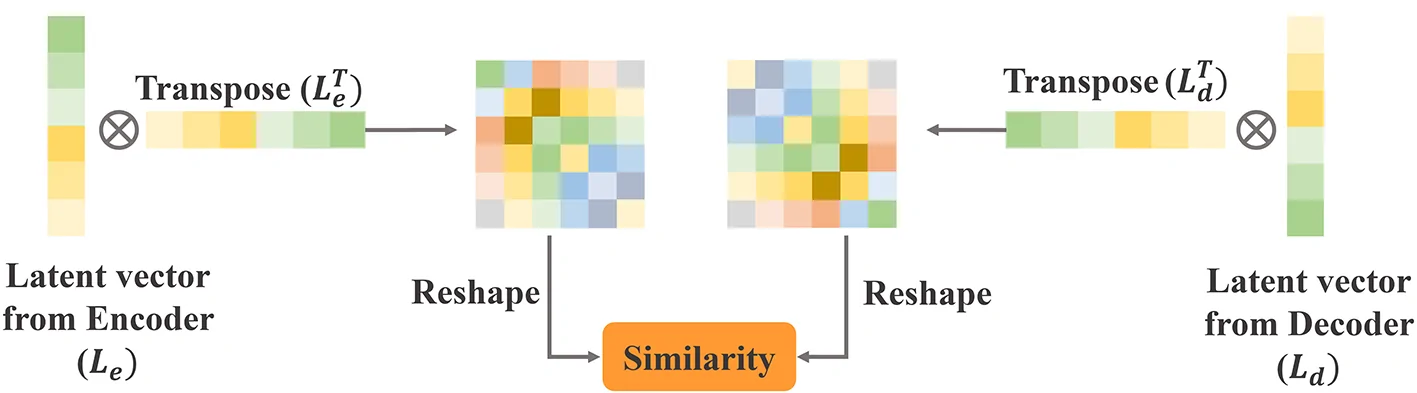
The structure of the FSD module. The symmetric latent vectors *L*_*e*/*d*_ are transposed ${(L\;}_{e/d}^{T})$ and multiply the original *L*_*e*/*d*_. We reshape *L*_*tra*_ into a 1-D vector (*L*_1−*D*_). And then, the feature similarity is obtained from the two 1D vectors.

In the pre-training stage, we independently train the baseline teacher and student sub-networks until parameter convergence, to obtain well-initialized junior models with stable feature extraction capabilities and reliable latent distribution priors. In the formal distillation optimization stage, we fix the backbone parameters of *T* and *S* and exclusively optimize the trainable parameters of the shared MLP projection heads by minimizing the feature reconstruction loss $L_{Rec}$. Statistically, $L_{Rec}$ quantifies the numerical discrepancy and distribution divergence of feature reconstruction between the projected latent embeddings of teacher features and student features in the unified latent space. Formally, the reconstruction loss $L_{Rec}$ is defined as follows Equation 5:


$$\begin{array}{l} {\;L}_{Rec}=||Fea_{in}-Fea_{out}{||}_{1} \end{array}$$ (5)

where *Fea*_*in*_ denotes the intrinsic features from *T* and *S*, and *Fea*_*out*_ illustrates the corresponding reconstructed features via the MLP projection heads. Furthermore, the designed feature-level similarity distillation is visualized in Figure 6. Specifically, the symmetric latent vectors $L_{e},L_{d}=\left[l_{1},l_{2},\cdots \;,l_{c}\right]\in R^{1\;\times \;512}$ are transposed ${(L\;}_{e}^{T}\;and{\;L\;}_{d}^{T})$ and multiplied by the original feature maps *L*_*e*_ *and L*_*d*_, yielding the intermediate response map *L*_*tra*_(512 × 512). Meanwhile, we reshape *L*_*tra*_(512 × 512) into a 1D vector (*L*_1−*D*_). Subsequently, we compute the cosine similarity to construct the feature-dimensional similarity maps. The cosine similarity metric is formally defined as follows Equation 6:

**Figure 6:**
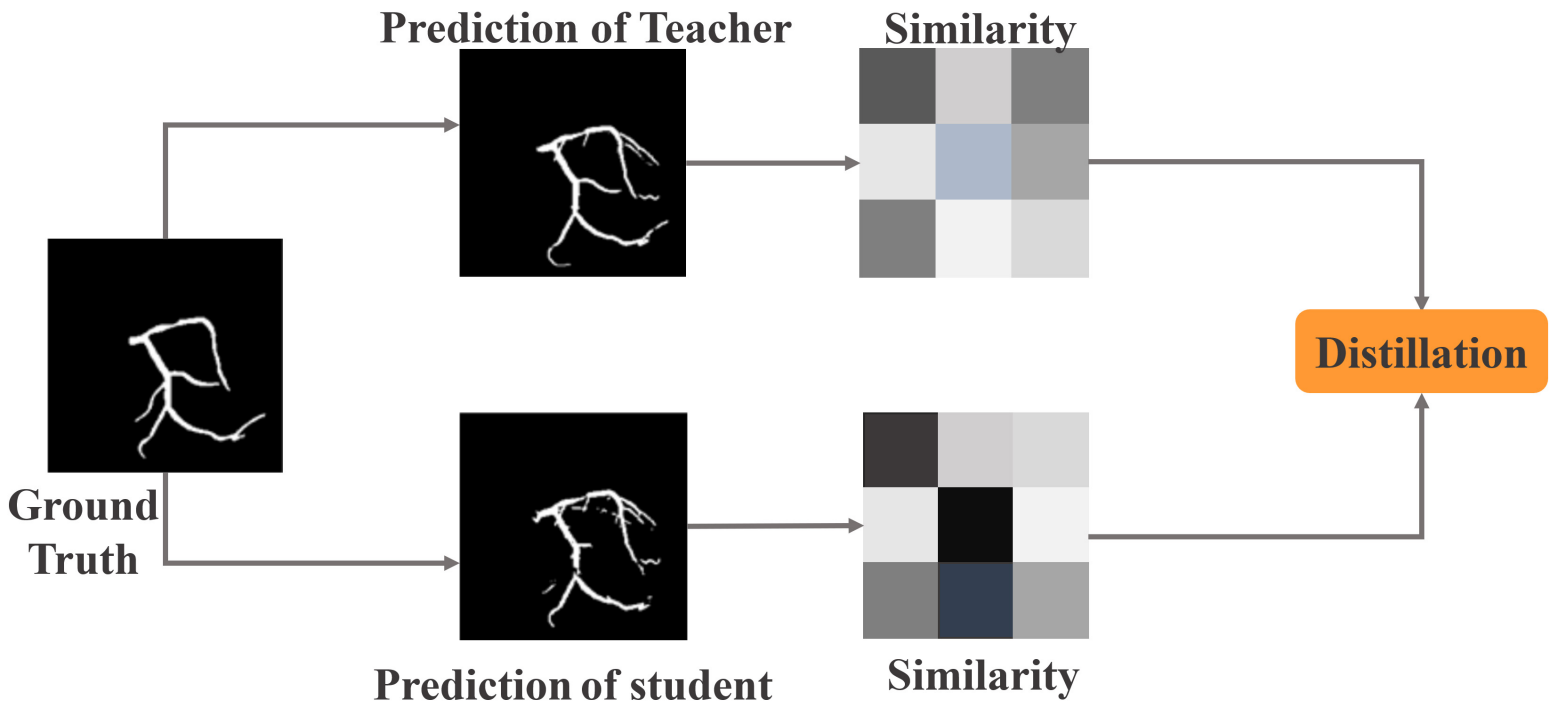
The structure of the ASD module. The ASD module imposes supervision on the spatial correlation between network predictions and ground-truth to achieve pixel-wise feature alignment.


$$\begin{array}{l} Cos\left(L_{e},L_{d}\right)=\frac{L_{e}\cdot L_{d}}{||L_{e}{||}_{2}\cdot ||L_{d}{||}_{2}} \end{array}$$ (6)

Here, *L*_*e*_ and *L*_*d*_ represent the feature latent vectors derived from the encoder and decoder, respectively. We calculate the pairwise cosine similarity among these latent features to explicitly characterize the semantic changes and transitions from the encoder to the decoder of *T* and *S*. Given the cosine similarity maps of the corresponding paired feature dimension from *T* and *S*, we employ a feature-level similarity constraint loss $L2\;\left(L_{FSD}\right)$ to enforce consistency between them. Formally, this loss can be formulated as Equation 7:


$$\begin{array}{l} {\;L}_{FSD}=||Cos_{tea}-Cos_{stu}{||}_{2} \end{array}$$ (7)

##### 3.4.1.3 Student refinement sub-network

As the pre-trained teacher refinement sub-network is only an ideal state and only available during the training phase, we hope that the student refinement sub-network can learn the knowledge from the teacher network and can be applied to both the training and testing phases. We implement a knowledge distillation strategy using FSD, and the student refinement sub-network is distilled by the teacher refinement sub-network. Specifically, the parameter-efficient student refinement sub-network is also constructed based on the U-Net (Ronneberger et al., 2015) backbone, which stacks inverted bottleneck block from MobileNetV2 (Sandler et al., 2018) within the encoder. MobileNetV2 squeeze the input channels down to 1/6 of their original demension, followed 3 × 3 depthwise separable convolutions, and ultimately performs linear projection for output compression. This architecture substantially reduces the channel dimensionality of intermediate layers.

The input of teacher refinement sub-network is combined original images *X* and the new gererated samples *X*_*new*_. But for student, its input is only original images *X*. So the student sub-network should be connected to improve the quality of segmented images in testing stage. In order to focus on the key areas and improve the efficiency of knowledge transfer, we use the FSD module as the transfer medium of knowledge between the teacher and student sub-network.

The designed teacher and student sub-networks have the different structures and inputs, which are different from the most existing knowledge distillation models where they usually adopt same inputs. The purpose of our framework is to make full use of the available images during teacher training, and distill the learned knowledge to student in testing when there are no generated images. Thus the special teacher-student design can achieve the purpose without high computational cost.

### 3.5 Pixel-level similarity distillation module

To further empower the lightweight student refinement sub-network *S* to learn more fine-grained semantic knowledge from the teacher refinement sub-network *T*, we introduce the PSD module as illustrated in Figure 7. Concretely, we first calculate the Euclidean distance *Euc*(*T, Z*) to evaluate pixel-level similarity between the predicted results and ground truth from the teacher model. Meanwhile, the Euclidean distance *Euc*(*S, Z*) between the prediction of student and ground truth is also obtained. The results from *Euc*(*T, Z*) and *Euc*(*S, Z*) construct pixel-level similarity semantic space ℝ. They are defined as follows Equations 8 and 9:


$$\begin{array}{l} \;Euc_{T}\left(T,Z\right)=\sqrt{\overset{n}{\underset{i=1}{\sum }}{\left(T_{i}-Z_{i}\right)}^{2}} \end{array}$$ (8)

$$\begin{array}{l} \;Euc_{S}\left(T,Z\right)=\sqrt{\overset{n}{\underset{i=1}{\sum }}{\left(S_{i}-Z_{i}\right)}^{2}} \end{array}$$ (9)

**Figure 7:**
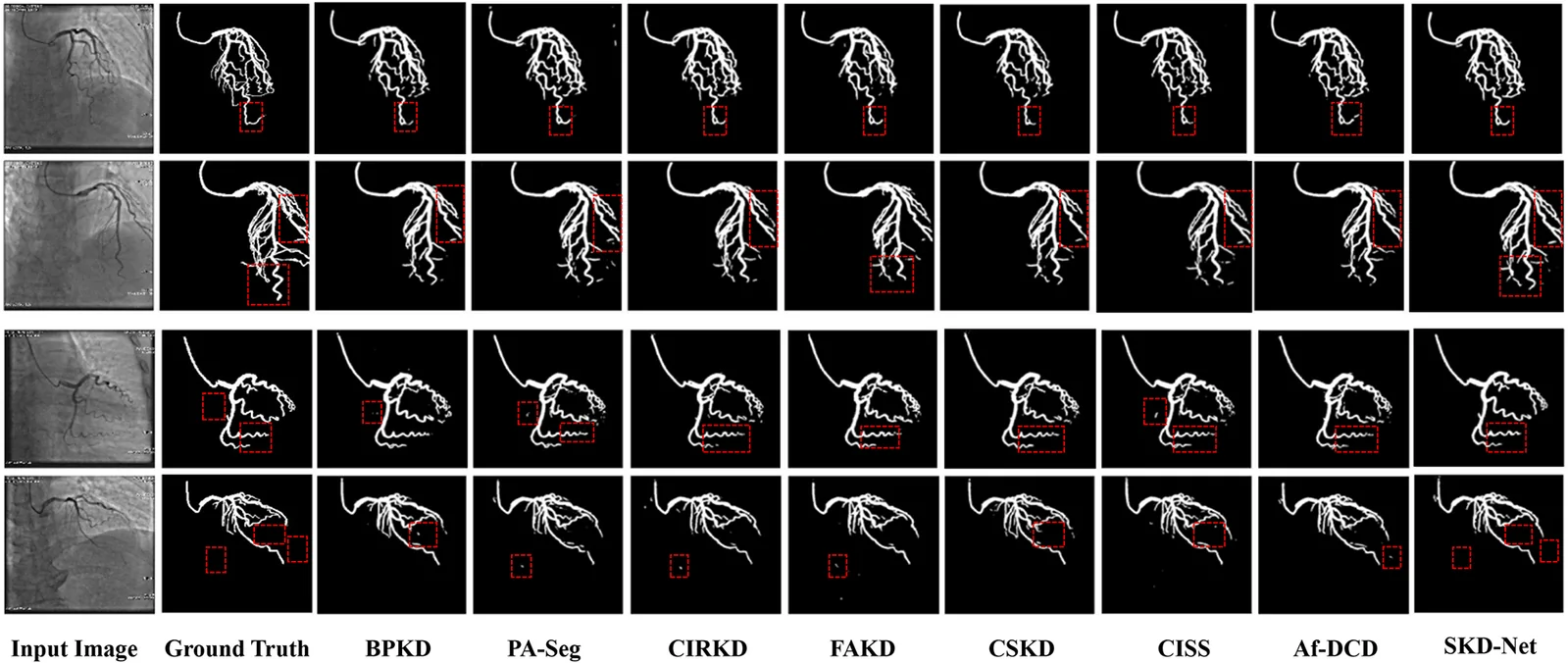
The visualization of quantitative comparison of SOTA knowledge distillation counterparts. Zoom in for better details.

where *T* illustrates the prediction maps of the teacher, *S* is the predicted results of the student, and *Z* represents the ground truth. Consequently, to enforce effective pixel-wise knowledge distillation from the complex teacher *T* to compact student *S*, we introduce a pixel-level constraint loss $L2\left(L_{PSD}\right)$. This loss is specifically designed to align the pixel-wise semantic distributions of *Euc*_*T*_ and *Euc*_*S*_, ensuring the student inherits fine-grained spatial and semantic knowledge. It is defined as follows Equation 10:


$$\begin{array}{l} {\;L}_{PSD}=||Euc_{T}-Euc_{S}{||}_{2} \end{array}$$ (10)

Additionally, adopting the standard cross-entropy loss $L_{CE}$ to supervise the training of the student model *S*. Specifically, this loss function is established by quantifying the discrepancy between the predictions and the corresponding ground-truth from the student model, serving as a fundamental supervision signal to guide the student to converge to accurate segmentation results. It is defined as Equation 11:


$$\begin{array}{l} {\;L}_{CE}=-\frac{1}{N}\left(y_{i}log\left(\bar{y_{i}}\right)+\left(1-y_{i}\right)log\left(1-\bar{y_{i}}\right)\right) \end{array}$$ (11)

where *y*_*i*_ shows ground-truth, $\bar{y_{i}}$ represents the predictions from the student model. *N* describes the total number of training samples. Finally, the overall optimization objective $L_{total}$ is formulated by integrating the cross-entropy loss $L_{CE}$, the feature-level similarity constraint loss $L_{FSD}$, and the pixel-level similarity loss $L_{PSD}$, which jointly guides the student refinement sub-network to learn both high-level semantic knowledge and fine-grained spatial details from the teacher network. Formally, the overall loss is expressed as Equation 12:


$$\begin{array}{l} L_{total}=L_{CE}+{\alpha L}_{FSD}+{\beta L}_{PSD}+\underset{G,G_{new}}{min}\underset{D}{max}L_{o}(G,G_{new},D) \end{array}$$ (12)

where α, and β are two hyper-parameters that balance the proportions between the two distillation losses. The overall training procedure pseudocode of KDLight is summarized in Algorithm 1.

Algorithm 1 Training procedure pseudocode of KDLight. 
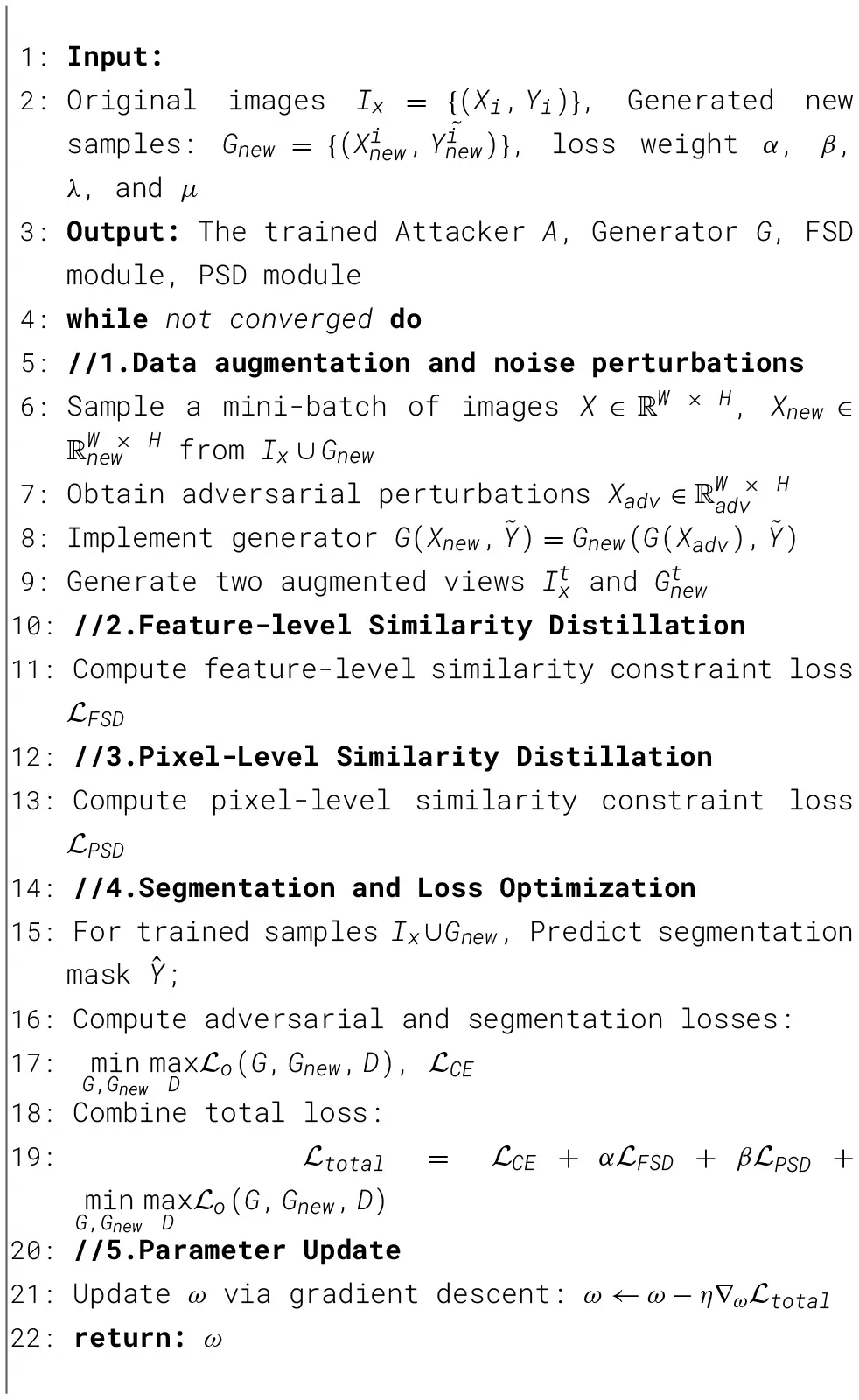

## 4 Experiments

### 4.1 Datasets and evaluation metric

#### 4.1.1 Datasets

In this study, experiments are conducted on two X-ray coronary angiography sequences datasets: Dataset 1 and Dataset 2. Dataset 1 (Javed, 2023) is a publicly available dataset with clinically sourced angiography images. The present database consists of 130 X-ray coronary angiograms from the Mexican Social Security Institute, and their corresponding ground-truth image outlined by an expert cardiologist. The ethics approval for its use in the present research, under reference R-2019-1001-078. Each angiogram is a 300 x 300 pixels, gray-scale image in pgm (Portable gray map) format. The image pixel spacing is mostly 0.37 mm/pixel. The Dataset 2 is collected from clinical Hospital. It contains 1415 angiography sequences collected from 486 subjects with coronary heart disease. The image sizes are 992 × 992, with pixel spacings of 0.20 mm/pixel. For Dataset 2, the total number of images is 7824, with each angiography sequence ranging from 5 to 27 frames in length. Each patient in both datasets has multiple angiography sequences captured from different viewing angles, selected by experienced radiologists to be either optimal or suboptimal for visualizing vessel morphology. The ground truth for the main coronary artery segmentation is provided by three experienced radiologists.

#### 4.1.2 Data preprocessing

The model first implement common data augmentation strategies on unlabeled coronary angiographic images, including random horizontal and vertical image flipping. It generates two differently augmented views of each original image. Note that different patients have different vessel sizes, and the vessels are often smaller in proportion to the image of a patient. If the entire image is directly into the network yields massive non-vessel background pixels, which impairs training convergence. To mitigate this problem, we crop each vessel image using its bounding cube and eliminate all out-of-cube background regions. The cube size is empirically fixed at 90 × 90. Moreover, we randomly extract (64 × 64) volumes from the selected cube region (90 × 90) of each images for data augmentation in the network training. It is important to note that we use the vessel mask solely to identify the location of coronary artery vessels. The input cube fed into the network contains not only the vessel region but also surrounding background areas.

#### 4.1.3 Evaluation metrics

We carry out various evaluation metrics to verify the coronary vessel segmentation performance of the proposed KDLight, reporting accuracy (ACC.), sensitivity (Sens.), specificity (Spec.), AUC (the area under the receiver operating characteristic curve), mean intersection over union (mIOU), and F1-score. Each is defined as follows Equations 13–17:

Accuracy: This metric quantifies the ratio of accurately predicted pixels to the total number of pixels.


$$\begin{array}{l} Accuracy=\frac{TP+TN}{TP+FP+TN+FN} \end{array}$$ (13)

where *TP* means true positives (correctly segmented pixels of this segment), *TN* represents true negative (correctly identified non-segment pixels), *FP* is false positive (incorrectly segmented pixels of this segment), and *FN* represents false negative (missed pixels of this segment).

Sensitivity (or recall): this measures the ratio of correctly segmented pixels for a category to the total number of ground truth pixels for that category.


$$\begin{array}{l} Sensitivity=\frac{TP}{TP+FP} \end{array}$$ (14)

Specificity: this metric measures the ratio of correctly predicted negative samples to the total number of actual negative samples.


$$\begin{array}{l} Specificity=\frac{TN}{TN+FP} \end{array}$$ (15)

The sensitivity and specificity are two important reference indices in the medical field, which measure the probability of missing and misdiagnosing patients.

Mean intersection over union (mIoU): IoU is calculated as the intersection ratio of the predicted vessel segmentation results for each class against the ground truth for that class. mIOU is the ratio of the intersection and union of all classes. It is employed to measure the extent of overlap between the prediction and the corresponding annotations.


$$\begin{array}{l} mIOU=\frac{1}{k+1}\;\times \;\frac{TP}{TP+FP+FN} \end{array}$$ (16)

where *k* represents the class, and (*k* + 1) represents the addition of the background.

F1-score: the F1-score provides a harmonic mean of precision and recall, providing a more balanced assessment of model performance. F1-score serves as an effective metric for evaluating segmentation performance in class-imbalanced scenarios.


$$\begin{array}{l} F1--score=\frac{2TP}{2TP+FP+FN}=\frac{2\cdot Presicion\cdot Sensitivity}{Presicion+Sensitivity} \end{array}$$ (17)

Moreover, to evaluate the lightweight efficiency of the proposed framework, we employ floating-point operations (FLOPs) and network parameter count (Params) as quantitative metrics.

### 4.2 Implementation details

The model is implemented in Python using the PyTorch (Paszke et al., 2017) deep learning framework and trained and evaluated on an NVIDIA RTX 3090 GPU. The number of training epochs is set to 200, with a batch size of 24. The weights of the loss function components are determined via grid search: the adversarial losses weight (λ, μ) is chosen from [0.05, 0.1, 0.3] and [0.05, 0.1, 0.5], feature-level similarity constraint loss weight (α) from [0.1, 0.3, 0.5, 1], and pixel-level similarity constraint loss weight (β) from [0.1, 0.3, 0.5, 1]. When trained on clinical coronary angiography data, the network adopts weights pre-trained on medical image datasets to accelerate convergence and improve model generalization.

We strictly adopt patient-independent five-fold cross-validation for all experiments, and the 80% data is implemented within each fold to standardize our training and evaluation pipeline. The remaining 20% data is used to test the proposed model. In addition, all reported mean and standard deviation values in our experimental results are calculated across the five cross-validation folds. This fold-wise statistical calculation ensures the reliability and robustness of our experimental results.

### 4.3 Experimental results

First, we conduct a comparative analysis between KDLight and several advanced approaches for coronary artery vessel segmentation, including the quantitative and qualitative result analysis in different datasets. We conducted a comprehensive review of recent literature on coronary artery segmentation. In these papers, we selectively included eight state-of-the-art methods (Liu et al., 2023, 2024; Zhai et al., 2025; Yang et al., 2025; Yuan et al., 2024; Karine et al., 2026; Baek et al., 2025; Fan et al., 2025) for comparison. All comparative methods are carefully re-implemented strictly following their original publications, covering baseline model, training strategy, and optimizer configuration. Furthermore, the effectiveness of Attacker, MGA, FSD, and PSD is comprehensively validated through extensive quantitative evaluations. Meanwhile, feature visualization is conducted to intuitively illustrate the working mechanism of these important modules.

#### 4.3.1 Comparison against the state-of-the-art methods

This section presents the comparison results of our proposed method against diverse SOAT knowledge distillation approaches, including StructKD (Liu et al., 2023), BPKD (Liu et al., 2024), PA-Seg (Zhai et al., 2025), CIRKD (Yang et al., 2025), FAKD (Yuan et al., 2024), CSKD (Karine et al., 2026), CISS (Baek et al., 2025), and Af-DCD (Fan et al., 2025). All experiments are conducted with consistent initial parameters to ensure the validity and comparability of the results. The quantitative experiment results are depicted in Tables 1, 2.

**Table 1:** The comparisons with SOTA knowledge distillation methods on Dataset 1 and Dataset 2.

Models	Acc. (%) ↑		Sens. (%) ↑		Spec. (%) ↑		AUC (%) ↑		mIOU (%) ↑		F1-score (%) ↑	
	Dataset 1	Dataset 2	Dataset 1	Dataset 2	Dataset 1	Dataset 2	Dataset 1	Dataset 2	Dataset 1	Dataset 2	Dataset 1	Dataset 2
**Teacher**	**98.42** **±1.22**	**97.68** **±0.75**	**90.83** **±1.75**	**88.66** **±0.59**	**78.52** **±0.44**	**77.69** **±1.93**	**97.24** **±1.18**	**97.77** **±0.24**	**88.85** **±1.69**	**86.91** **±1.81**	**87.64** **±0.79**	**86.25** **±1.29**
Stu.-scratch	96.45 ± 0.42	94.63 ± 0.04	84.72 ± 0.34	83.62 ± 0.72	75.45 ± 4.41	73.38 ± 3.20	94.84 ± 0.14	94.71 ± 0.35	86.30 ± 1.84	83.47 ± 1.74	81.18 ± 1.83	79.47 ± 0.43
StructKD	97.03 ± 0.78	95.48 ± 0.56	84.39 ± 0.24	85.67 ± 1.09	76.40 ± 0.73	74.32 ± 0.44	97.75 ± 1.75	96.23 ± 0.07	86.47 ± 2.40	82.85 ± 1.35	82.47 ± 0.63	80.23 ± 0.38
BPKD	97.84 ± 1.03	96.07 ± 0.21	87.12 ± 0.19	87.03 ± 0.70	75.82 ± 0.35	76.03 ± 1.57	98.31 ± 0.88	95.83 ± 0.01	85.37 ± 0.45	85.47 ± 0.59	82.75 ± 0.72	81.46 ± 0.46
PA-Seg	95.39 ± 0.46	96.30 ± 0.03	85.40 ± 2.56	86.39 ± 0.36	75.40 ± 2.12	74.63 ± 2.54	97.35 ± 0.08	96.36 ± 0.06	86.21 ± 0.26	84.32 ± 0.64	83.52 ± 0.84	79.50 ± 3.27
CIRKD	96.94 ± 0.21	97.03 ± 0.52	86.59 ± 0.46	85.92 ± 0.45	76.68 ± 3.95	75.21 ± 1.56	97.50 ± 0.43	96.23 ± 0.18	85.41 ± 0.52	81.60 ± 0.26	81.90 ± 0.40	81.85 ± 0.72
FAKD	98.02 ± 0.56	95.39 ± 0.43	86.32 ± 0.08	86.53 ± 0.17	76.73 ± 1.84	76.49 ± 0.56	97.28 ± 0.02	96.47 ± 0.53	86.35 ± 0.21	84.42 ± 0.38	79.94 ± 0.67	80.35 ± 1.08
CSKD	98.16 ± 0.09	96.31 ± 0.05	87.49 ± 1.73	86.90 ± 0.64	77.04 ± 4.63	76.48 ± 2.16	96.84 ± 0.17	96.54 ± 0.06	85.94 ± 0.36	80.94 ± 0.54	82.46 ± 3.84	80.75 ± 0.17
CISS	97.52 ± 0.31	96.38 ± 0.67	87.17 ± 0.27	86.84 ± 0.37	76.43 ± 0.85	75.38 ± 0.63	97.85 ± 0.72	95.67 ± 0.03	86.57 ± 0.53	85.35 ± 0.26	83.57 ± 1.60	79.08 ± 0.53
Af-DCD	97.43 ± 0.05	96.16 ± 0.50	87.94 ± 0.54	85.30 ± 0.74	76.52 ± 2.75	76.68 ± 0.79	97.26 ± 0.96	95.73 ± 0.11	86.40 ± 0.63	84.74 ± 1.60	82.45 ± 3.06	80.41 ± 0.42
**Our method**	**98.39** **±0.15**	**97.52** **±1.04**	**90.81** **±0.84**	**88.19** **±0.16**	**78.89** **±1.96**	**77.14** **±1.72**	**97.83** **±0.38**	**96.94** **±0.08**	**89.04** **±0.49**	**86.35** **±0.52**	**88.47** **±1.46**	**86.61** **±2.19**

All of these methods are trained and evaluated on two datasets using a five-fold cross-validation. And these metrics are averaged across all branches to evaluate the overall performance. ↑ indicates that the higher is better. ↑ denotes standard deviations. Stu.-scratch denotes the student network without EMA, FSD, and PSD modules. The bold values (Teacher and Our method) denote the experimental results from teacher network in KDLight and proposed KDlight.

**Table 2:** The detailed *p*-values of all performances in two datasets.

Models	Acc. (%) ↑		Sens. (%) ↑		Spec. (%) ↑		AUC (%) ↑		mIOU (%) ↑		F1-score (%) ↑	
	Dataset 1	Dataset 2	Dataset 1	Dataset 2	Dataset 1	Dataset 2	Dataset 1	Dataset 2	Dataset 1	Dataset 2	Dataset 1	Dataset 2
BPKD	0.0013	0.0042	0.0315	0.0006	0.0008	0.0182	0.0005	0.0002	0.0001	0.0021	0.0007	0.0300
PA-Seg	0.0008	0.0005	0.0068	0.0045	0.0067	0.0380	0.0006	1.28 × 10^−8^	0.0009	0.0045	0.0076	0.0007
CIRKD	2.07 × 10^−11^	0.0087	0.0241	0.0074	0.0004	0.0311	0.0001	0.0095	0.0060	0.0300	0.0033	0.0075
FAKD	0.0001	0.0080	0.0345	0.0001	0.9 × 10^−6^	0.0037	0.2303	0.0003	0.0002	0.0004	3.29 × 10^−12^	0.0001
CSKD	0.0070	0.0009	0.0475	0.0007	0.0009	0.0179	0.0025	0.0008	4.38 × 10^−11^	0.0006	0.0006	0.0076
CISS	0.0051	0.0006	3.85 × 10^−7^	0.0008	0.0009	0.0006	0.0017	0.0062	0.0067	0.0051	0.0003	0.0029
Af-DCD	0.0439	0.0005	0.0475	0.0090	0.0004	0.0228	0.0004	0.0278	0.0003	0.0001	0.0067	0.0106

The *p*-values indicate the significance of the differences between the proposed method and other models.

##### 4.3.1.1 Experiments on the Dataset 1

Results, shown in Table 1, report significantly higher performance by our proposed KDLight compared with SOTA knowledge distillation counterparts in Dataset 1. Concretely, our approach shows a great gain in the accuracy, sensitivity, specificity, AUC, mIOU, and F1-score. Particularly, the sensitivity and specificity are the most significant metrics for clinical validation of automated angiography analysis tools. The two performance metrics gained by 2.87 (KDLight 90.81 vs. Af-DCD 87.94) and 1.81 (KDLight 78.49 vs. Af-DCD 76.68), respectively. Then, the mIOU increases by 1.17 (KDLight 87.74 vs. CISS 86.57), which demonstrates that KDLight improves the overlapping degree of the coronary vessel segmentation compared with ground truth. Remarkably, our proposed method enables the KDLight to achieve competitive performance compared with the teacher model in terms of the specificity and mIOU in PCAD (i.e., Spec.: KDLight 78.89 vs. Teacher 78.52, mIOU: KDLight 89.04 vs. Teacher 88.85). Additionally, compared with the original student network and SOTA knowledge method, our proposed method achieves significant performance improvements without introducing additional computational overhead. Specifically, Table 2 illustrates the quantitative comparison of lightweight evaluation indices in Dataset 1. It is observed that the FLOPs and Params. from the teacher model are 354.34G and 130.85M without knowledge distillation. However, the performance drops to 21.31 and 14.62 when the knowledge distillation is implemented.

##### 4.3.1.2 Experiments on the Dataset 2

Table 1 also displays the superiority of KDLight in comparison with. The SOTA counterparts in Dataset 2. Similarly, all the performance metrics obtain a noticeable growth compared with various knowledge distillation networks and Stu.-scratch. Specifically, the accuracy obtained a gain of 0.49% (KDLight 97.52% vs. CIRKD 97.03%). The sensitivity and specificity increase by 1.16 (KDLight 88.19% vs. BPKD 87.03%) and 0.46% (KDLight 77.14% vs. Af-DCD 76.68%), respectively. The performances of AUC and mIOU have achieved a gain of 0.4% (KDLight 96.94% vs. CSKD 96.54%) and 0.88% (KDLight 86.35% vs. BPKD 85.47%) for vessel segmentation. Importantly, the performance of the F1-score has exceeded that of the teacher network, achieving 86.61%.

Furthermore, statistical significance testing with *p*-values consistently smaller than 0.05 on the two datasets confirms the superior performance of the proposed method compared to other state-of-the-art models in Table 2. The lightweight performance metrics have obtained remarkable progress, as depicted in Table 3. The detailed FLOPs and Params are 21.31G (vs. Teacher 354.34) and 14.62M (vs. Teacher 130.85) on two datasets.

**Table 3:** The lightweight comparisons with SOTA knowledge distillation methods on Dataset 1 and Dataset 2.

Models	FLOPs (G) ↓		Params. (M) ↓	
	Dataset 1	Dataset 2	Dataset 1	Dataset 2
**Teacher**	**354.34**	**354.34**	**130.85**	**130.85**
Stu.-scratch	35.63	35.63	20.74	20.74
StructKD	48.86	48.86	32.43	32.43
BPKD	29.06	29.06	20.18	20.18
PA-Seg	27.48	27.48	19.62	19.62
CIRKD	35.11	35.11	27.57	27.57
FAKD	25.45	25.45	18.47	18.47
CSKD	27.93	27.93	19.16	19.16
CISS	24.12	24.12	17.38	17.38
Af-DCD	26.83	26.83	16.48	16.48
**Our method**	**21.31**	**21.31**	**14.62**	**14.62**

All of these methods are trained and evaluated on two datasets using a five-fold cross-validation. And these metrics are averaged across all branches to evaluate the overall performance. ↓ indicates that the lower is better. The bold values (Teacher and Our method) denote the experimental results from teacher network in KDLight and proposed KDLight.

##### 4.3.1.3 Better visualization results in representative images

We also show the visual comparison results predicted by comparative knowledge distillation counterparts and our KDLight, illustrated in Figure 8. Notably, the coronary artery images exhibit extremely complex vascular textures, which pose significant challenges to accurate segmentation. While most existing knowledge distillation methods struggle to effectively segment the vessel foreground from background pixels, our proposed method consistently outperforms these counterparts in coronary vessel segmentation tasks. Quantitative and qualitative results demonstrate that KDLight can extract vascular regions that are more consistent with the annotations compared to the SOTA approaches under such complex vascular scenarios.

**Figure 8:**
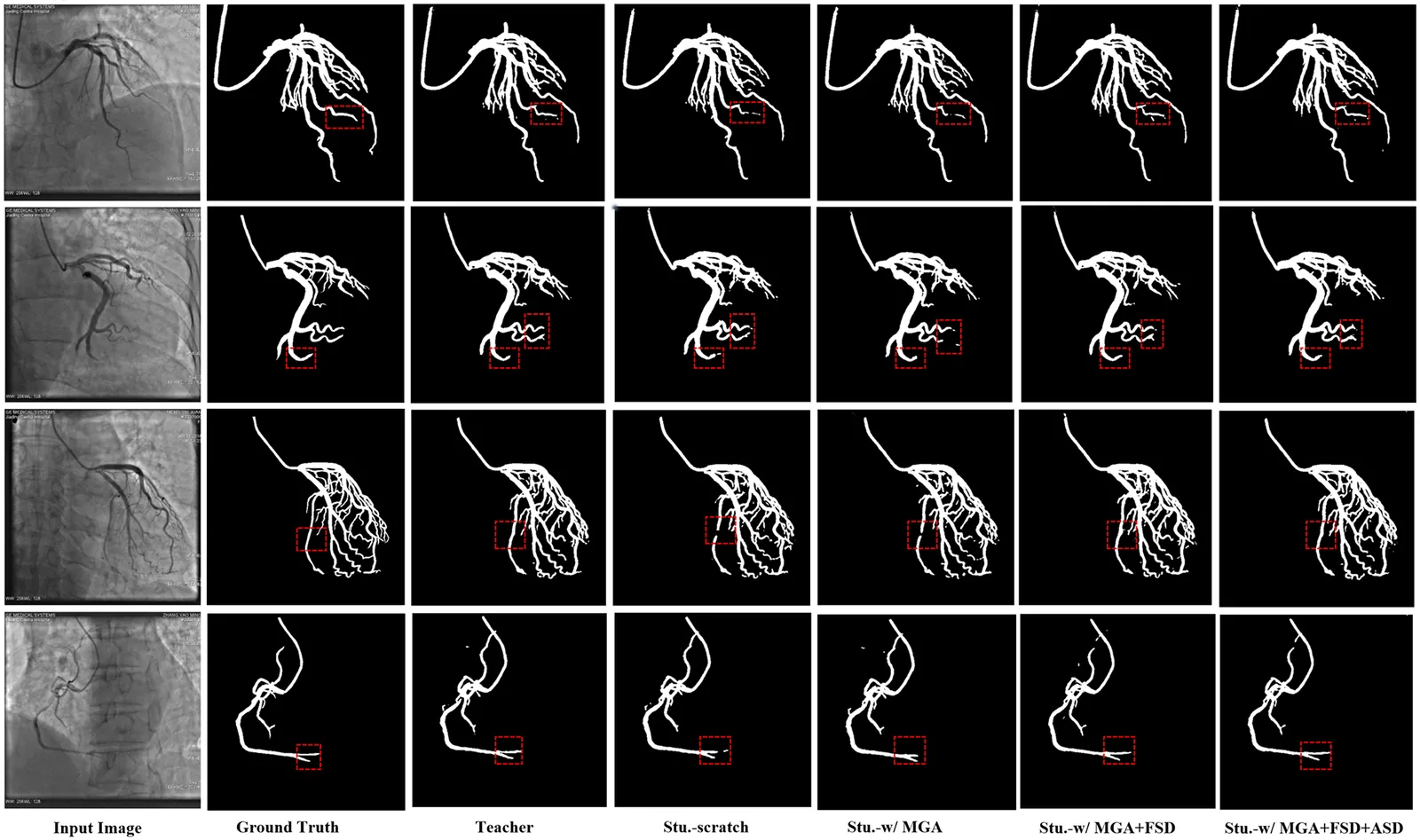
The visualization compares segmentation results for ablation analysis. Zoom in for better details.

As evidenced by the above experimental results, the KDLight not only yields higher overlap between segmentation predictions and annotations, but also delineates vessel boundaries with significantly greater precision and robustness.

##### 4.3.2 Effectiveness evaluation of the ablation study

In the following experiments, we further conduct ablation studies to validate our architectural design, including the MGA, FSD, PSD, and Adversarial Attacker modules. It presents the ablation study results through different combinations of three modules in the KDLight. For brevity, we also report the experimental results over the Dataset 1 and Dataset 2, respectively.

##### 4.3.2.1 The effectiveness of MGA module

In the experiment, we first denote the coarse vessel segmentation as Stu.-scratch. And then, we show the effectiveness of MGA modules in Table 4 (Stu. w/MGA). As shown, we quantitatively assess the contribution of the proposed MGA schema to the vessel segmentation task. Compared with Stu.-scratch, the proposed KDLight with MGA module improves these performances on the Dataset 1 and Dataset 2, which further validates the high efficiency of the MGA. These results indicate that the MGA effectively enhances feature discriminability, particularly in suppressing background false positives. MGA achieves this improvement at minimal computational cost, validating its role as a lightweight yet effective feature enhancer.

**Table 4:** The comparative results of the ablation study.

Models	Acc. (%) ↑		Sens. (%) ↑		Spec. (%) ↑		AUC (%) ↑		mIOU (%) ↑		F1-score (%) ↑	
	Dataset 1	Dataset 2	Dataset 1	Dataset 2	Dataset 1	Dataset 2	Dataset 1	Dataset 2	Dataset 1	Dataset 2	Dataset 1	Dataset 2
**Teacher**	**98.42** **±1.22**	**97.68** **±0.75**	**90.83** **±1.75**	**88.66** **±0.59**	**78.52** **±0.44**	**77.69** **±1.93**	**97.24** **±1.18**	**97.77** **±0.24**	**88.85** **±1.69**	**86.91** **±1.81**	**87.64** **±0.79**	**86.25** **±1.29**
Stu.-scratch	94.89 ± 0.31	93.12 ± 1.42	85.86 ± 1.05	85.73 ± 2.36	74.81 ± 1.61	75.02 ± 0.47	94.39 ± 1.55	94.37 ± 1.37	84.10 ± 0.98	83.77 ± 0.39	82.15 ± 1.25	80.33 ± 0.70
Stu. w/MGA	95.07 ± 1.66	93.88 ± 2.11	86.29 ± 0.62	86.22 ± 1.67	76.95 ± 0.73	75.43 ± 1.55	95.68 ± 0.88	94.92 ± 2.05	84.97 ± 1.38	84.58 ± 1.11	84.07 ± 0.68	81.86 ± 1.97
Stu. w/MGA + FSD	96.83 ± 0.93	94.26 ± 0.33	86.88 ± 1.87	86.80 ± 0.92	77.53 ± 1.14	75.96 ± 1.18	96.16 ± 1.33	95.63 ± 0.66	85.36 ± 0.22	85.04 ± 0.97	85.28 ± 1.09	83.15 ± 0.29
Stu. w/MGA + FSD + PSD	97.23 ± 1.42	96.35 ± 1.86	87.60 ± 1.29	87.54 ± 1.25	77.67 ± 0.73	76.50 ± 0.81	96.48 ± 0.57	96.40 ± 1.49	87.14 ± 1.82	85.69 ± 1.63	85.95 ± 2.08	83.72 ± 1.52
**Stu. w/AA** **+** **MGA** **+** **FSD** **+** **PSD**	**98.39** **±0.15**	**97.52** **±1.04**	**90.81** **±0.84**	**88.19** **±0.16**	**78.49** **±1.96**	**77.14** **±1.72**	**97.83** **±0.38**	**96.94** **±0.08**	**87.74** **±0.49**	**86.35** **±0.52**	**87.47** **±1.46**	**85.61** **±2.19**

We illustrate the functional effectiveness of each module, including MGA, FSD, and PSD modules. In an ablation study, the Dataset 1 and Dataset 2 are combined and fed to these models. ↑ indicates that the higher is better. ± scratch denotes the student network without Adversarial Attacker module (AA), MGA, FSD, and PSD modules. The bold values (Teacher and Stu. w/AA+MGA+FSD+PSD) denote the experimental results from teacher network in KDLight and proposed KDLight.

##### 4.3.2.2 The effectiveness of FSD module

In the meantime, as reported in Table 4 (Stu. w/MGA + FSD), compared with the baseline Stu. w/MGA, the network with MGA and FSD achieves significant improvement in all the metrics. Concretely, the accuracy grows by 1.76% and 0.38%, and F1-score also grows by 1.21% and 1.29% on both datasets. Other performance have also been improved. The FSD transfers intermediate representation knowledge from the teacher, enabling the student to capture richer structural patterns. The stable improvement across both datasets confirms that FSD effectively bridges the encoder–decoder feature gap and aligns the student's feature distribution with that of the teacher.

##### 4.3.2.3 The effectiveness of PSD module

Moreover, as depicted in Table 4 (Stu.w/MGA + FSD + PSD), the proposed KDLight, along with three modules, performs better than the model with MGA and FSD modules (Stu.w/MGA + FSD). Specifically, PSD contributes the most substantial gain in mIOU (1.78% on Dataset 1) and a notable accuracy grows by 2.09% on Dataset 2. FSD enforces fine-grained spatial correspondence, which directly benefits pixel-wise segmentation precision. The complementary nature of FSD (semantic-level) and PSD (spatial-level) is evident that their combination outperforms either distillation paradigm alone, justifying the dual-level distillation design.

##### 4.3.2.4 The effectiveness of adversarial attacker module

The adversarial attacker module delivers the most comprehensive performance leap. Sensitivity surges by 3.21% on Dataset 1, Accuracy improves by 1.16% and 1.17%, and F1-score gains 1.52% and 1.89% across both datasets. The adversarial training paradigm forces the student to produce more robust and clinically consistent predictions, especially in identifying vessel foreground regions. With adversarial attacker integrated, the lightweight student model approaches even exceeds the teacher's performance on Dataset 1(AUC 97.83% vs. 97.24%), demonstrating that adversarial attacker is the key component closing the teacher–student performance gap.

#### 4.3.3 Generalization study on different student networks

The student network plays a pivotal role in our proposed framework. In practical deployment scenarios, the student network is required to be lightweight to achieve fast inference speed, while simultaneously retaining the high segmentation accuracy inherited from the teacher network. Our shallow student network is constructed based on the popular U-Net. In this study, we construct different student models to show the comparison results to demonstrate the generalization of KDLight. Specifically, KDLight is compared against a diverse set of baseline methods, including the classical segmentation networks U-Net (Ronneberger et al., 2015), U-Net++ (Zhou et al., 2018), and U-Net 3+ (Huang et al., 2020), the popular light-weight network Enet (Paszke et al., 2016), MobileNetv3 (Andrew et al., 2019), and ERFNet (Romera et al., 2018).

As illustrated in Table 5, the experimental results clearly indicate that KDLight outperforms all other student network counterparts. This result indicates that our proposed method exhibits stronger generalization capability across various student network architectures. Notably, the FLOPs and Params of KDLight are substantially lower than those of the teacher network, confirming its parameter efficiency while maintaining high performance.

**Table 5:** The generalization experiment on different student networks.

Models	Acc. (%) ↑		Sens. (%) ↑		Spec. (%) ↑		AUC (%) ↑		mIOU (%) ↑		F1-score (%) ↑	
	Dataset 1	Dataset 2	Dataset 1	Dataset 2	Dataset 1	Dataset 2	Dataset 1	Dataset 2	Dataset 1	Dataset 2	Dataset 1	Dataset 2
**Teacher**	**98.42** **±1.22**	**97.68** **±0.75**	**90.83** **±1.75**	**88.66** **±0.59**	**78.52** **±0.44**	**77.69** **±1.93**	**97.24** **±1.18**	**97.77** **±0.24**	**88.85** **±1.69**	**86.91** **±1.81**	**87.64** **±0.79**	**86.25** **±1.29**
St.-UNet-Scratch	91.38 ± 1.47	90.27 ± 0.43	82.41 ± 2.19	83.72 ± 0.39	72.49 ± 0.32	72.62 ± 1.04	95.43 ± 0.12	96.39 ± 2.01	83.59 ± 0.37	84.38 ± 2.51	76.39 ± 1.03	77.49 ± 0.35
St.-UNet-Our	95.47 ± 0.86	96.39 ± 0.05	85.28 ± 1.80	85.28 ± 0.46	74.37 ± 0.14	74.37 ± 2.59	97.55 ± 0.03	98.27 ± 1.37	85.03 ± 1.45	87.04 ± 1.38	78.67 ± 2.10	80.27 ± 1.05
St.-UNet++-Scratch	93.23 ± 0.43	93.73 ± 2.20	84.07 ± 0.27	83.91 ± 0.28	73.29 ± 1.04	74.08 ± 0.32	95.50 ± 0.42	96.72 ± 0.24	83.27 ± 0.32	84.74 ± 1.96	78.97 ± 0.42	78.93 ± 1.03
St.-UNet++-Our	95.26 ± 0.54	96.29 ± 1.36	86.36 ± 0.47	87.84 ± 1.39	76.20 ± 2.39	77.38 ± 0.53	96.74 ± 1.49	99.58 ± 1.36	85.84 ± 0.53	87.63 ± 1.04	80.43 ± 0.15	82.69 ± 1.78
St.-UNet3+-Scratch	92.04 ± 1.39	89.92 ± 1.54	84.92 ± 0.15	85.29 ± 2.40	75.39 ± 0.34	74.64 ± 0.50	94.79 ± 1.37	95.83 ± 2.42	82.39 ± 0.51	83.98 ± 0.53	80.37 ± 0.35	80.37 ± 1.65
St.-UNet3+-Our	96.76 ± 1.40	97.35 ± 1.50	88.72 ± 0.42	89.05 ± 1.39	78.21 ± 2.03	78.38 ± 0.14	97.37 ± 0.34	98.64 ± 1.47	86.97 ± 0.63	88.03 ± 0.75	82.49 ± 0.53	85.29 ± 0.003
St.-ENet-Scratch	90.07 ± 0.03	89.61 ± 0.47	81.92 ± 0.39	82.19 ± 1.07	71.54 ± 2.45	72.48 ± 0.27	93.68 ± 0.73	94.37 ± 1.37	83.03 ± 1.03	82.74 ± 0.14	76.73 ± 0.45	79.47 ± 0.68
St.-ENet-Our	93.18 ± 0.79	92.84 ± 0.68	86.83 ± 0.16	85.29 ± 1.44	73.48 ± 1.49	76.30 ± 1.58	96.75 ± 0.41	97.05 ± 1.03	85.48 ± 1.53	86.49 ± 1.74	79.63 ± 0.53	82.53 ± 0.45
St.-MobileNetv3-Scratch	92.37 ± 1.44	90.18 ± 0.91	80.37 ± 0.47	79.49 ± 2.08	72.08 ± 0.48	73.38 ± 1.24	92.70 ± 1.03	94.67 ± 1.42	82.58 ± 1.28	83.48 ± 1.53	78.38 ± 1.59	80.57 ± 0.42
St.-MobileNetv3-Our	94.28 ± 2.01	93.72 ± 1.07	85.36 ± 1.37	84.47 ± 1.38	75.47 ± 0.63	78.59 ± 0.53	95.83 ± 1.53	98.30 ± 0.34	84.73 ± 1.53	88.34 ± 1.24	80.79 ± 0.48	82.81 ± 0.51
St.-ERFNet-Scratch	89.03 ± 1.06	88.38 ± 1.36	82.47 ± 1.47	83.62 ± 0.28	74.23 ± 0.29	72.45 ± 0.17	94.73 ± 1.05	94.29 ± 0.28	83.39 ± 1.64	82.57 ± 1.20	79.87 ± 0.74	79.08 ± 1.04
St.-ERFNet-Our	92.38 ± 0.46	92.02 ± 1.65	87.92 ± 0.46	88.73 ± 0.04	77.82 ± 1.57	76.83 ± 0.02	96.57 ± 0.37	98.37 ± 0.74	86.05 ± 1.04	87.73 ± 0.53	81.58 ± 1.05	83.56 ± 1.64

↑ indicates that the higher is better.

#### 4.3.4 Applicability and effectiveness assessment on cross-domain segmentation

We conduct cross-domain segmentation experiments on two disparate datasets to further substantiate the robustness and generalization performance of KDLight. Concretely, we adopt a cross-domain training-testing paradigm where the Dataset 2 is used for model training, while the Dataset 1 dataset is employed for performance evaluation. Conversely, we train on Dataset 1 and test on Dataset 2. As illustrated in Table 6, in contrast to the results of training and testing within the same dataset (i.e., 1th, 2th, and 3th rows), when training set is Dataset 2 and testing set is Dataset 1 (5th row), the accuracy decrease. The other performance indices have also decreased to varying degrees. The visualization results are illustrated in Figure 9. We observe that the Dataset 2 and Dataset 1 exhibit substantial domain discrepancies, stemming from differences in data sources and imaging scanners. Additionally, Dataset 1 is a public dataset with annotations restricted to main vessel branches, while the Dataset 2 undergoes exhaustive and fine-grained annotations for the contrary vessel. Although the performance of our KDLight slightly declines in different domains, it still demonstrates the competitive applicability and effectiveness on cross-domain contrary artery vessel segmentation.

**Table 6:** The effectiveness of cross-domain segmentation.

Models	Training Dataset	Testing Dataset	Acc. (%)	Sens. (%)	Spec. (%)	AUC (%)	mIOU (%)	F1-score (%)
Our method	Dataset 1	Dataset 1	98.59 ± 0.20	88.03 ± 0.32	77.81 ± 1.36	99.13 ± 0.19	88.24 ± 0.56	84.76 ± 0.65
	Dataset 2	Dataset 2	98.45 ± 0.17	87.94 ± 0.50	78.05 ± 0.84	98.68 ± 0.06	87.15 ± 0.88	81.96 ± 0.2
	Dataset 1 + Dataset 2	Dataset 1 + Dataset 2	97.64 ± 0.74	88.27 ± 0.52	77.82 ± 0.06	98.52 ± 0.48	87.66 ± 0.27	85.20 ± 0.54
	Dataset 1	Dataset 2	89.38 ± 1.03	78.36 ± 0.26	72.16 ± 1.63	92.42 ± 0.59	80.72 ± 0.42	81.07 ± 1.37
	Dataset 2	Dataset 1	94.47 ± 0.18	83.81 ± 0.57	74.54 ± 2.42	95.03 ± 1.04	84.71 ± 1.38	84.37 ± 2.16

**Figure 9:**
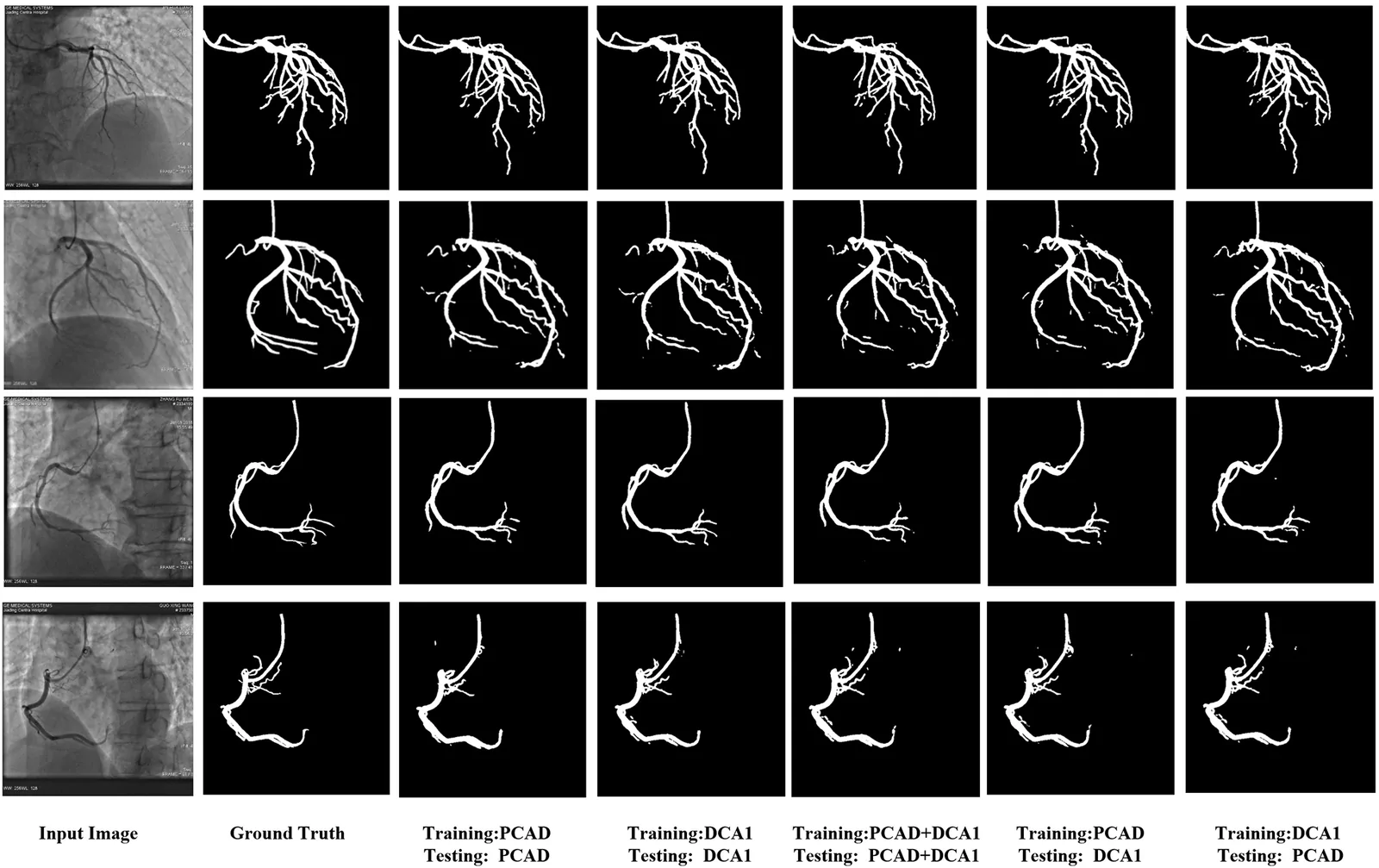
The cross-domain visualization segmentation results. Overall, the results indicate that our SKD-Net possesses excellent generalization capabilities, performing robustly on cross-domain data for segmentation.

#### 4.3.5 t-SNE visual verification of KDLight

To comprehensively validate the effectiveness of KDLight, we implement the t-SNE visualization on the latent vectors of the student-scratch (w/o KD) and student network (w/KD). As displayed in Figure 10, the powerful student network with KD performs a clear distribution of discrepancies upon the anchor samples and various augmented samples. The scratch-trained student network without KDLight shows non-cluster impact. Furthermore, in our KDLight framework, we propose an FSD and PSD distillation schema to model the learning of generalizable knowledge via feature similarity and spatial adversarial correlation. Once the student obtains knowledge from the teacher, the latent vectors demonstrate noticeable distribution discrepancies compared with scratch-trained ones. This demonstrates that our KDLight can enforce the student network to be more robust and generalizable.

**Figure 10:**
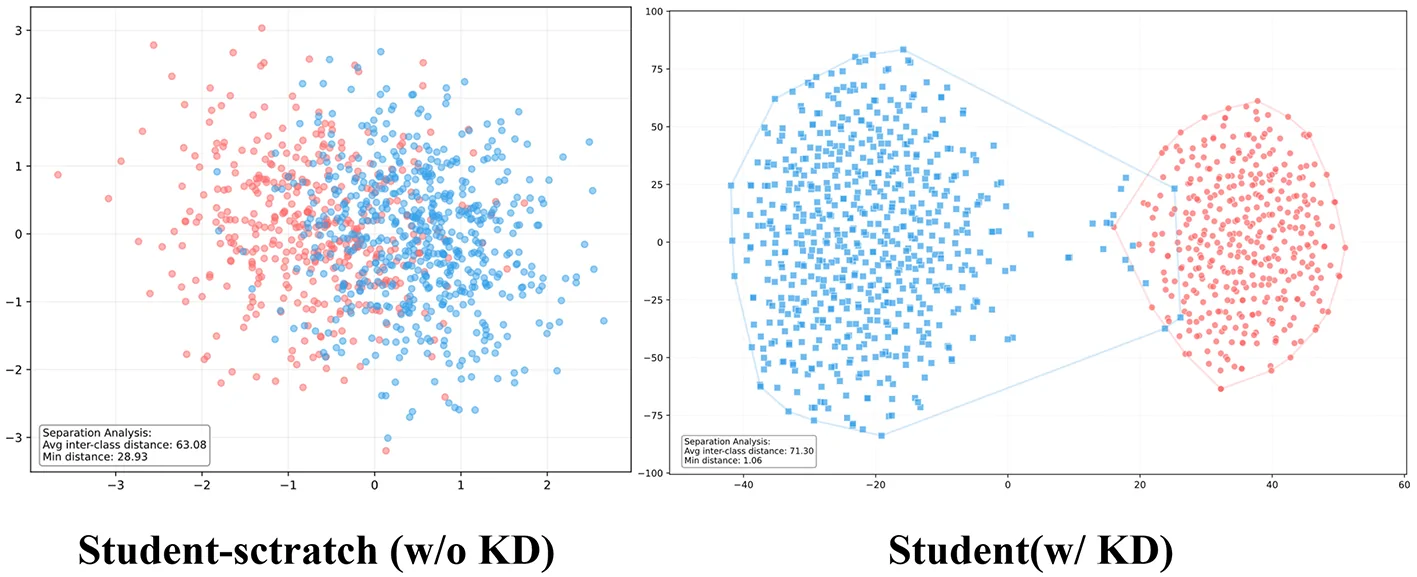
T-SNE visualization on the latent vectors of student networks with SKD-Net. Upon knowledge distillation, the latent vectors of the student model exhibit significant distributional discrepancies compared with scratch-trained counterparts. Zoom in for better details.

#### 4.3.6 Heatmap visualization based on Grad-CAM

To verify the interpretability of our KDLight, we utilize Grad-CAM to visually interpret the decoder module within the student network. The heatmaps produced by the decoder allow us to visualize the attention distribution across regions during segmentation inference, clearly revealing its decision-making process. This visualization capability facilitates researchers in better understanding the underlying learning rules of the model and attributing the discriminative features learned by the model to specific vascular regions. Figure 11 presents the heatmaps generated by the student network before and after the application of knowledge distillation. It can be clearly observed that without knowledge distillation, the attention heatmaps output by the student network are highly ambiguous, especially in the task of distinguishing vascular edges from the background (consistent with the coronary vessel segmentation scenario). In contrast, after incorporating knowledge distillation, the student model precisely focuses on the target vascular regions while significantly reducing its attention to irrelevant background areas. This observation demonstrates that the proposed knowledge distillation strategy allows the student to better grasp core requirements of the vessel segmentation task, effectively enhances its segmentation performance, and suppresses the model's attention to non-discriminative, irrelevant information.

**Figure 11:**
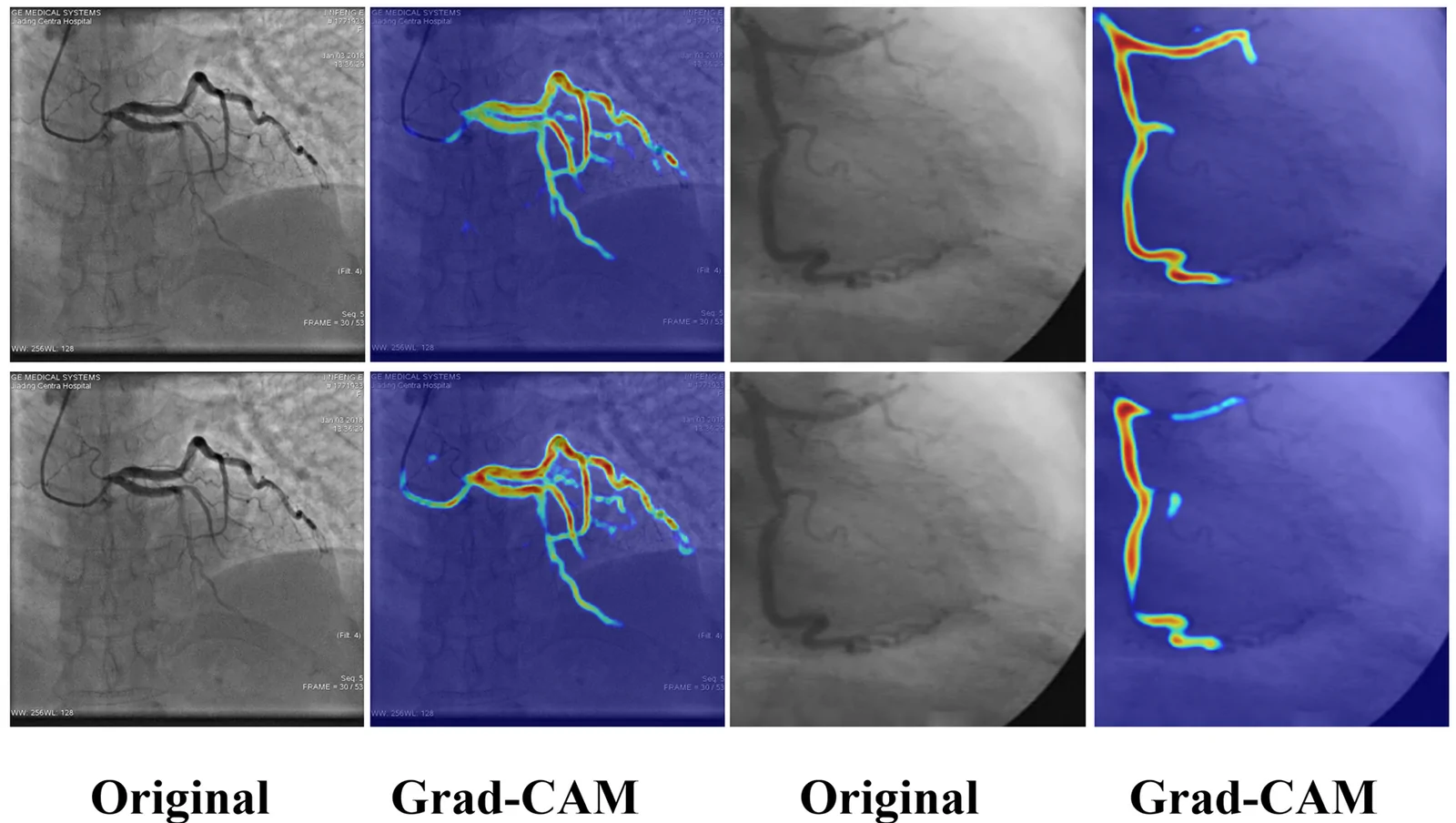
Gradient-weighted class activation mapping.

## 5 Discussion

In this work, we propose KDLight, a lightweight knowledge distillation framework embedded with a black-box adversarial attacker for coronary artery vessel segmentation on X-ray coronary angiography images. We design four core components: the MGA, FSD, PSD, and the adversarial attacker modules. Ablation studies on two independent datasets with distinct imaging characteristics and annotation standards verify the individual and joint contributions of each component.

The MGA module serves as a lightweight feature enhancement block for the teacher refinement sub-network. By grouping channel features and fusing multi-scale spatial information, it reduces computational overhead while preserving cross-channel and cross-spatial semantic dependencies. Compared with the scratch-trained student baseline, introducing MGA brings consistent improvements in specificity, mIOU and F1-score on both Dataset 1 and Dataset 2, which demonstrates its capacity to suppress background false-positive responses and highlight vascular foreground regions. Nevertheless, relying solely on MGA cannot sufficiently narrow the performance gap between the heavy teacher and compact student, since the student still lacks high-level semantic knowledge learned by the teacher network.

To transfer hierarchical semantic knowledge from teacher to student, we develop the FSD module, which aligns encoder-decoder feature distributions via cosine similarity constraints in a shared latent embedding space. After integrating FSD upon MGA, all segmentation metrics are further boosted. FSD focuses on global semantic feature matching, yet it cannot fully supervise fine-grained pixel-level prediction discrepancies, especially for tiny coronary branches with blurred boundaries. For this reason, we introduce the complementary PSD module. PSD builds pixel-wise similarity space based on Euclidean distances between network predictions and ground-truth labels, forcing the student to mimic the teacher's fine spatial reasoning. The obvious gain of mIOU after adding PSD confirms that dual-level distillation combining FSD and PSD is more effective than single feature-oriented distillation for vessel segmentation tasks.

The black-box adversarial attacker module further improves model robustness and segmentation performance. It generates diverse realistic adversarial perturbations including Gaussian noise, impulse noise and misalignment artifacts, simulating real-world interference factors in X-ray angiography acquisition. Under adversarial training, the student achieves substantial sensitivity improvements and its AUC even surpasses the teacher on Dataset 1. This reveals that adversarial perturbation feedback effectively mitigates overfitting caused by limited clinical annotated samples. However, we also observe performance degradation in cross-domain validation. As shown in cross-domain experiments (Table 5), when training and testing datasets come from different medical centers with divergent imaging parameters and annotation granularity, all metrics drop to varying degrees. Domain shift remains a non-negligible limitation for practical clinical deployment.

Comparative experiments against recent state-of-the-art knowledge distillation segmentation methods demonstrate that KDLight achieves competitive segmentation accuracy with remarkably reduced FLOPs and parameter counts. Generalization experiments over multiple student backbones verify that our distillation pipeline can be migrated to different lightweight encoder architectures. Still, there are several directions deserving further exploration in future work. First, we will collect more multi-center angiography data to alleviate domain gap and improve cross-dataset generalization. Second, the current framework focuses on binary vessel segmentation; extending it to multi-class segmentation for different coronary branches and automatic stenosis quantification will bring higher clinical practicability. Third, we will optimize the inference pipeline to further reduce latency, facilitating real-time auxiliary diagnosis in interventional catheter-lab scenarios.

## 6 Conclusion

This paper presents an efficient and systematic knowledge distillation method tailored for heart vessel segmentation, termed KDLight. The framework presents three efficient methods: the Adversarial Attacker module, the Multi-Scale Group Attention (MGA), the Feature-level Similarity Distillation module (FSD), and the Pixel-Level Similarity Distillation module (PSD). Specifically, we first utilize a black-box attacker to generate adversarial examples. This adversarial process not only prevents from overfitting but also enhances the robustness of the model for image segmentation. MGA is designed to preserve the channel-wise information of complex coronary artery vessels while effectively reducing computational overhead. Through the design of multi-scale and group attention mechanisms, MGA effectively captures both short-term and long-range dependencies, as well as extracts cross-channel and cross-spatial feature interactions, which are critical for accurate vessel segmentation. The FSD aims to strengthen the semantic feature extraction ability of the student network by aligning the feature distributions of the teacher and student models. In parallel, the PSD introduces the pixel-level semantic information from the annotations to optimize the fine-grained prediction accuracy of the student model, which effectively alleviates the ambiguity issue in small vessel segmentation. Extensive experimental evaluations validate that KDLight achieves superior performance over state-of-the-art knowledge distillation approaches on coronary artery vessel segmentation tasks.

## Data Availability

The datasets presented in this study can be found in online repositories. The names of the repository/repositories and accession number(s) can be found at: http://personal.cimat.mx:8181/~ivan.cruz/DB_Angiograms.html.
