# Supplementary material for: Exploring lightweight knowledge distillation with adversarial attack for efficient coronary artery vessel segmentation
Source: Front Artif Intell. 2026 Oct 30;9:1891993. doi: 10.3389/frai.2026.1891993 (PMC13759833; doi:10.3389/frai.2026.1891993)
Supplement: Supplementary file 1 [file Supplementary_file_1.docx]

Supplementary Material

# 1 Supplementary Figures and Tables

## 1.1 Supplementary Figures

## **Supplementary Figure 1.** The coronary angiographic image specimens. Red bounding boxes denote the complex background and noise of angiography images. Zoom in for better details.

**Supplementary Figure 2.** The framework of our proposed KDLight. It mainly includes three modules, namely Adversarial Attacker Module on the left. Meanwhile, the Feature-level Similarity Distillation Module on the Middle, the Pixel-Level Similarity Distillation Module on the right.

**Supplementary Figure 3.** The perturbation samples. (a) Gaussian, (b) Impulse, (c)Misalign, and (d) Uniform.

**Supplementary Figure 4.** Illustration of the Multi-Scale Group Attention module. Here, *K* denotes the number of divided groups, *X Avg Pool* refers to the one-dimensional horizontal global average pooling, and $X_{new}$ *Avg Pool* represents the one-dimensional vertical global average pooling, respectively. *Matmul* means Matrix Multiplication.

**Supplementary Figure 5.** The structure of the MLP module. The contextual feature maps are mapped into a latent feature space via two Multi-Layer Perceptron (MLP) with GELU activation, which performs linear projection, non-linear transformation, and a second linear projection. The MLP module is constructed to generate latent vectors. Finally, the semantic feature information before and after the hidden vector is reconstructed via the reconstruction loss ${\mathcal{\mathcal{L}}}_{\text{R}\text{ec}}$.

**Supplementary Figure 6.** The structure of the FSD module. The symmetric latent vectors $L_{e/d}$ are transposed ${(L}_{e/d}^{T})$ and multiply the original $L_{e/d}$. We reshape $L_{tra}$ into a 1-D vector $(L_{1-D})$. And then, the feature similarity is obtained from the two 1D vectors.

**Supplementary Figure 7.** The structure of the PSD module. The PSD module imposes supervision on the spatial correlation between network predictions and ground-truth to achieve pixel-wise feature alignment.

**Supplementary Figure 8.** The visualization of quantitative comparison of SOTA knowledge distillation counterparts. **Zoom in for better details.**

**Supplementary Figure 9.** The cross-domain visualization segmentation results. Overall, the results indicate that our KDLight possesses excellent generalization capabilities, performing robustly on cross-domain data for segmentation.

**Supplementary Figure 10.** T-SNE visualization on the latent vectors of student networks with KDLight. Upon knowledge distillation, the latent vectors of the student model exhibit significant distributional discrepancies compared with scratch-trained counterparts. **Zoom in for better details.**

**Supplementary Figure 11.** Gradient-weighted class activation mapping
